# Genome‐wide analyses of *Liberibacter* species provides insights into evolution, phylogenetic relationships, and virulence factors

**DOI:** 10.1111/mpp.12925

**Published:** 2020-02-28

**Authors:** Shree P. Thapa, Agustina De Francesco, Jessica Trinh, Fatta B. Gurung, Zhiqian Pang, Georgios Vidalakis, Nian Wang, Veronica Ancona, Wenbo Ma, Gitta Coaker

**Affiliations:** ^1^ Department of Plant Pathology University of California Davis CA USA; ^2^ Department of Microbiology and Plant Pathology University of California Riverside CA USA; ^3^ Citrus Center Department of Agriculture, Agribusiness and Environmental Sciences Texas A&M University‐Kingsville Weslaco TX USA; ^4^ Citrus Research and Education Center Department of Microbiology and Cell Science University of Florida Lake Alfred FL USA

**Keywords:** ‘*Candidatus* Liberibacter’ sp., citrus greening disease, HLB, huanglongbing, phylogenomics, SEC effector

## Abstract

‘*Candidatus* Liberibacter’ species are insect‐transmitted, phloem‐limited α‐Proteobacteria in the order of *Rhizobiales*. The citrus industry is facing significant challenges due to huanglongbing, associated with infection from ‘*Candidatus* Liberibacter asiaticus’ (Las). In order to gain greater insight into ‘*Ca.* Liberibacter’ biology and genetic diversity, we have performed genome sequencing and comparative analyses of diverse ‘*Ca*. Liberibacter’ species, including those that can infect citrus. Our phylogenetic analysis differentiates ‘*Ca.* Liberibacter’ species and *Rhizobiales* in separate clades and suggests stepwise evolution from a common ancestor splitting first into nonpathogenic *Liberibacter crescens* followed by diversification of pathogenic ‘*Ca.* Liberibacter’ species. Further analysis of Las genomes from different geographical locations revealed diversity among isolates from the United States. Our phylogenetic study also indicates multiple Las introduction events in California and spread of the pathogen from Florida to Texas. Texan Las isolates were closely related, while Florida and Asian isolates exhibited the most genetic variation. We have identified conserved Sec translocon (SEC)‐dependent effectors likely involved in bacterial survival and virulence of Las and analysed their expression in their plant host (citrus) and insect vector (*Diaphorina citri*). Individual SEC‐dependent effectors exhibited differential expression patterns between host and vector, indicating that Las uses its effector repertoire to differentially modulate diverse organisms. Collectively, this work provides insights into the evolution of ‘*Ca.* Liberibacter’ species, the introduction of Las in the United States and identifies promising Las targets for disease management.

## Graphical abstract

Comparative analyses of genomes provide insights into the evolution of ‘*Ca.* Liberibacter’ species and the introduction of huanglongbing in the United States, and identifies promising conserved Sec translocon‐dependent effector targets for disease management.

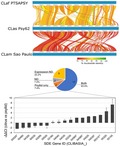

## 1 INTRODUCTION

Three ‘*Candidatus* Liberibacter’ species are associated with huanglongbing (HLB), which is also known as citrus greening disease: ‘*Ca.* Liberibacter asiaticus’ (Las), ‘*Ca.* Liberibacter africanus’ (Laf), and ‘*Ca.* Liberibacter americanus’ (Lam). Las, Laf, and Lam are insect‐transmitted, phloem‐limited, and unculturable in axenic culture (Bove, 2006). Las and Lam are transmitted by the Asian citrus psyllid (ACP) *Diaphorina citri*, while Laf is transmitted by the African citrus psyllid *Trioza erytreae* (Bove, 2006; Wang *et al.*, 2017). Las is the most widely distributed species, first reported in Asia, but has spread to Africa, the Middle East, as well as North, Central, and South America (Gotwald, 2010; Wang, 2019). In Africa and Madagascar, HLB is primarily associated with Laf and the disease occurs in cool areas, with temperatures below 30 °C (Catling, 1969; Schwarz and Green, 1972). Lam was found in Brazil but has been displaced by Las (Coletta‐Filho *et al*., 2010).

Las is the most common HLB‐associated bacterium, which is the most damaging disease of citrus worldwide (Gottwald *et al.*, 2007; Hodges *et al.*, 2015). The disease is characterized by blotchy mottling and yellowing of leaves, canopy defoliation and dieback, small deformed, unevenly coloured off‐tasting fruit, premature fruit drop, loss of fibrous roots, and overall root decline ultimately leading to tree death (Bove, 2006; Bassanezi *et al.*, 2009; Graham *et al.*, 2013).

HLB was first described in 1919 in Guangdong, China and remains an important disease in this country (Tu, 1932). HLB was reported in São Paulo, Brazil in 2004, but may have been there since the early 1990s (Teixeira *et al.*, 2005; Bove, 2006). In the United States, Las was detected in Florida in 2005 and has since rapidly spread to Texas, Louisiana, South Carolina, Georgia, and California (da Graça *et al.*, 2016). In Florida alone, HLB has resulted in over $7.8 billion in lost revenue and more than 7,500 jobs lost since 2007 (Hodges *et al.*, 2015). In 2017, the net orange production in Florida fell behind that of California for the first time in recent history, with a nearly two‐fold decrease in Florida production (http://agnetwest.com/california-citrus-production-outpaces-florida/). In Texas, Las was detected in 2012 and has spread in different counties; however, the impact of HLB is not as severe as in Florida (Sétamou *et al.*, 2019). In California, Las‐positive citrus was detected in 2012, but there were no subsequent positives until 2015 (Dai *et al.*, 2019). An increase in the number of positives has been observed in California, with 1,713 detections in the last 5 years (https://www.cdfa.ca.gov/plant/acp/). However, no Las‐positive citrus has been reported in commercial California groves, which support a $3.4 billion industry with a total economic impact of $7.1 billion (Babcock, 2018). It was recently estimated that a 20% reduction in California citrus acreage would cause a loss of 7,350 jobs and $127 million in employee income, and reduce state GDP by $501 million (Babcock, 2018). Management of the disease is not only difficult but also expensive, and no cure is available (Wang, 2019. Currently, the best preventative measures include chemical treatment for vector control, removal of infected trees, and replanting with healthy nursery stock (Belasque *et al.*, 2010; Wang, 2019).

In addition to the species associated with HLB on citrus, there are four additional ‘*Ca.* Liberibacter’ species. ‘*Ca.* Liberibacter solanacearum’ (Lso) is an emerging pathogen of potatoes, tomatoes, and carrots (Liefting *et al.*, 2009; Munyaneza *et al.*, 2010). Lso is transmitted to solanaceous and apiaceous plants with distinct psyllid species transmitting separate haplotypes to different hosts (Wang *et al.*, 2017). ‘*Ca.* Liberibacter europaeus’ (Leu) was first described as an endophyte of pear trees in Italy and is transmitted by the pear psyllid (*Cacopsylla pyri*) (Raddadi *et al.*, 2011). Later, Leu was shown to be associated with a disease on Scotch broom (*Cytisus scoparius*) (Thompson *et al.*, 2013). *Liberibacter crescens* (Lcr) and ‘*Ca.* Liberibacter brunswickensis’ (Lbr) are not known to be associated with plant disease (Raddadi *et al.*, 2011; Leonard *et al.*, 2012). Lbr was first identified in the Australian eggplant psyllid, *Acizzia solanicola* (Morris *et al.*, 2017). Lcr was first isolated from the phloem sap of a defoliated Babaco papaya, *Carica stipulata* × *Carica pubescens*, in Puerto Rico in 1995 (Leonard *et al.*, 2012). Lso, Leu, and Lbr are obligate intracellular pathogens, Lso and Leu have small genomes with size c.1.2 Mbp, but Lbr has not been sequenced yet (Lin *et al.*, 2011; PSQJ01000002). While Lcr is the only culturable *Liberibacter* species, additional plant hosts and its insect vector remain unknown (Jain *et al.*, 2019).

Las, Laf, and Lam are obligate intracellular pathogens and have relatively small genomes (c.1.2 Mb) (Duan *et al.*, 2009; Wulff *et al.*, 2014; Lin *et al.*, 2015). All three pathogens occur in a persistent, propagative, circulative manner within their insect vectors after acquisition during plant feeding (Gottwald, 2010). Las metabolism has been investigated, revealing it is not able to synthesize tryptophan, tyrosine, leucine, isoleucine, and valine from metabolic intermediates (Duan *et al.*, 2009; Wulff *et al.*, 2014). Las must counter these deficiencies by importing these amino acids from either its host or vector. Las can manipulate *Diaphorina citri's* metabolism in order to obtain energetic nucleotides and this results in a shorter lifespan and altered vector feeding behaviour (Killiny *et al.*, 2017).

Bacterial plant pathogens are known to have secreted proteins, called effectors, that play an essential role in their pathogenesis. Effectors from diverse pathogens aid infection by suppressing plant immunity and creating environments favourable for colonization and proliferation (Toruno *et al.*, 2016). Unlike most gram‐negative bacteria, Las lacks the type III secretion system, but possesses the general Sec secretion system, which is capable of secreting effectors directly outside bacterial cells (Sugio *et al.*, 2011). Sec translocon‐dependent effectors (SDEs) are among the most likely Las virulence components (Pagliaccia, *et al.*, 2017; Clark *et al.*, 2018). However, the host targets of Las SDEs remain relatively unexplored. One Las SDE, SDE1 (CLIBASIA_05315), is primarily expressed in citrus and acts to inhibit defence‐induced host papain‐like cysteine protease activity (Pagliaccia, *et al.*, 2017; Clark *et al.*, 2018).

Pathogenic species of ‘*Ca.* Liberibacter’ are difficult to study because of their inability to be cultured, their phloem‐limited nature, and their intracellular life in plant hosts. The genetic diversity of Las in different citrus‐growing regions in the United States is poorly understood and diversity studies have primarily focused on prophage sequences (Zhou *et al.*, 2013; Zheng *et al.*, 2017; Dai *et al.*, 2019). Whole‐genome sequences of Las collected in different locations will provide insight into the evolution, diversity, and introduction of Las. In this study, we analysed 36 *Liberibacter* genomes and eight other *Rhizobiales* to gain a greater understanding of this important genus. Evolutionary analyses indicate that ‘*Ca*. Liberibacter’ underwent a stepwise divergence toward pathogenicity. Phylogenetic analyses using core orthologous genes indicate that Las has been introduced multiple times in California. Investigation of potential virulence factors within citrus‐infecting liberibacters highlighted the presence of SDEs, which are widely conserved between Las isolates but not across all three species. SDEs were differentially expressed in their plant host and insect vector, indicating these effectors have specialized functions.

**Figure 1:**
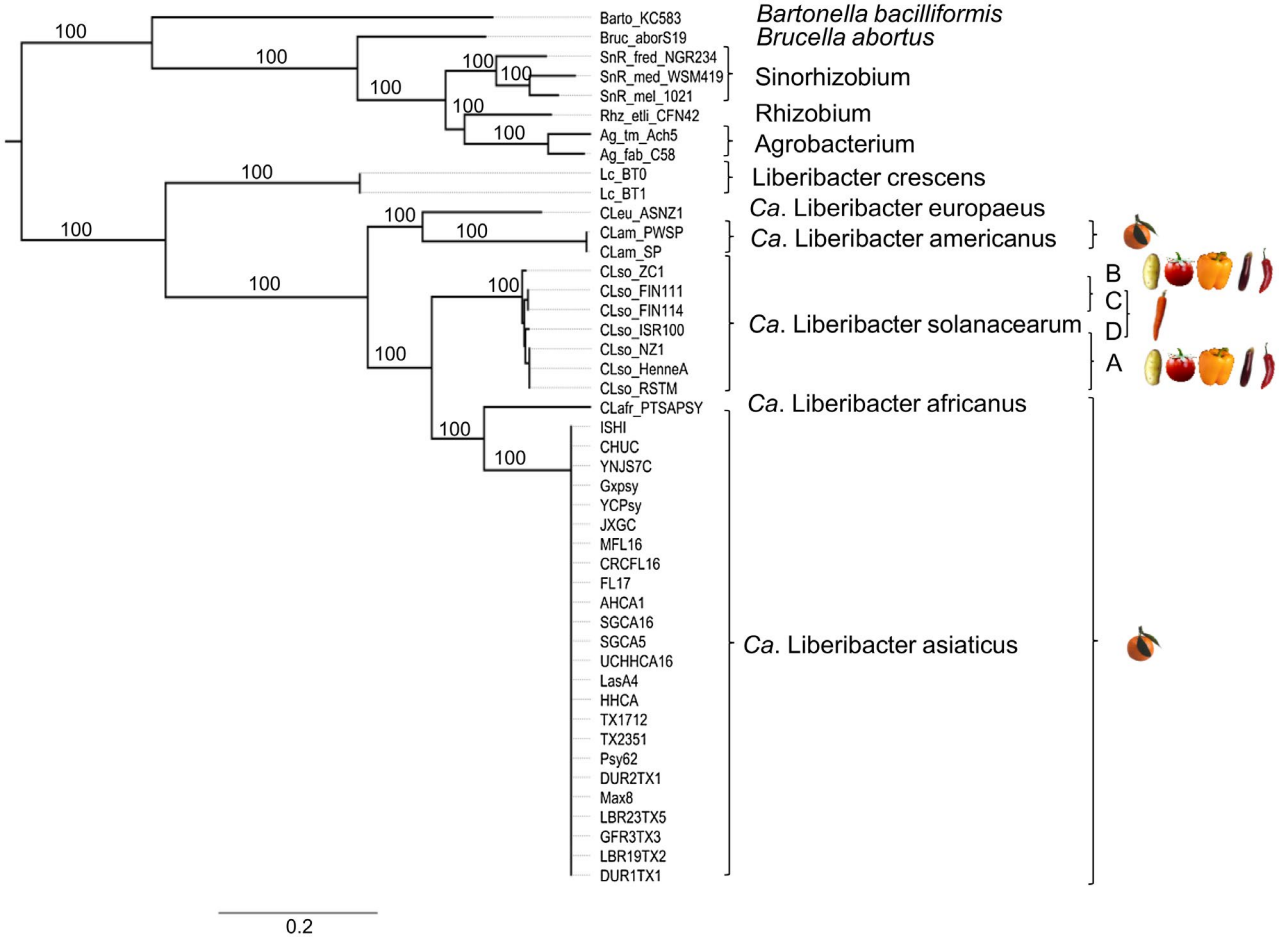
Genome‐wide comparison of species within the *Rhizobiales*. Maximum‐likelihood phylogeny of ‘*Ca.* Liberibacter’ species and other isolates from *Rhizobiales*. In total, 345 orthologous genes were concatenated. A maximum‐likelihood approach was used to generate the phylogeny with 1,000 bootstrap replicates. Bootstrap values are indicated at each node. To the right are images of crops infected by specific ‘*Ca.* Liberibacter’ species. The resulting phylogeny was visualized using FigTree v. 1.4.3 (Rambaut, 2012)

## 2 RESULTS

### 2.1 ‘*Ca.* Liberibacter’ species form a monophyletic group distinct from other *Rhizobiales*

‘*Ca.* Liberibacter’ is a member of the *Rhizobiales* and related to *Sinorhizobium*, *Rhizobium*, *Agrobacterium*, *Brucella*, and *Bartonella*. We sequenced and assembled 11 isolates of Las that were obtained from infected citrus in Florida (2), Texas (5), California (2), Mexico (1), and China (1) using a combination of Illumina HiSeq 2,500, Miseq, and TruSeq platforms. Samples from Florida, Texas, and Mexico were collected from commercial groves. Samples from California were obtained from Las‐infected trees from urban areas in the south part of the state. The isolate from China (CHUC) was intercepted by the Citrus Clonal Protection Program (CCPP, UC Riverside, National Clean Plant Network), in a variety introduced into California. We also analysed 13 sequences of Las available in the NCBI database from China, Japan, Texas, and California and included genomes of Lam (2), Laf (1), Lso (7), Leu (1), Lcr (2), and other *Rhizobiales* (8) (Table S1). Additional information for all isolates used in the study is summarized in Table S1.

We identified 345 orthologous genes across all tested genomes, which were concatenated for phylogenetic analyses using a maximum‐likelihood approach in RAxML v. 8.2 (Stamatakis, 2006). The resulting phylogeny clearly separates the ‘*Ca.* Liberibacter’ species from other *Rhizobiales* with high bootstrap support (Figure 1). The Lso isolates, which all cause disease in solanaceaous crops, cluster together in a single clade but the three species of citrus‐infecting *Liberibacter* do not: Las and Laf cluster together in one clade while Lam clusters with Leu in a separate clade. Lcr, the only culturable *Liberibacter* species, forms a distinct clade separated from all other *Rhizobiales* (Figure 1).

Whole‐genome comparisons using average nucleotide identity (ANI) demonstrate that the ‘*Ca*. Liberibacter’ species designations are accurate, with high ANI values within each species, but low ANI values between species (Figure S1). All Las isolates are closely related with ANIs of 99%–100% (Figure S1).

### 2.2 ‘*Ca*. Liberibacter’ species separated in a stepwise fashion

The disease chronology record suggests that Las was the first pathogen to appear on citrus. To obtain deeper insight into history and divergence times of different *Liberibacter* species, we reconstructed their phylogenetic relationships based on concatenated sequences of 345 core orthologous genes. Ambiguously aligned regions were removed using GBlocks (Castresana, 2000). Because of the lack of fossil data, our phylogenetic analyses are based on the time split of *Sinorhizobium*/*Agrobacterium* to calibrate the molecular clocks. The divergence times of *Sinorhizobium* and *Agrobacterium* were previously estimated to be 140.10–200.87 and 100.10–149.07 million years ago (mya), respectively (Chriki‐Adeeb and Chriki, 2016). The Bayesian tree was inferred using MrBayes v. 3.2 with Markov chain Monte Carlo runs for 10 million generations (Huelsenbeck and Ronquist, 2001).

**Figure 2:**
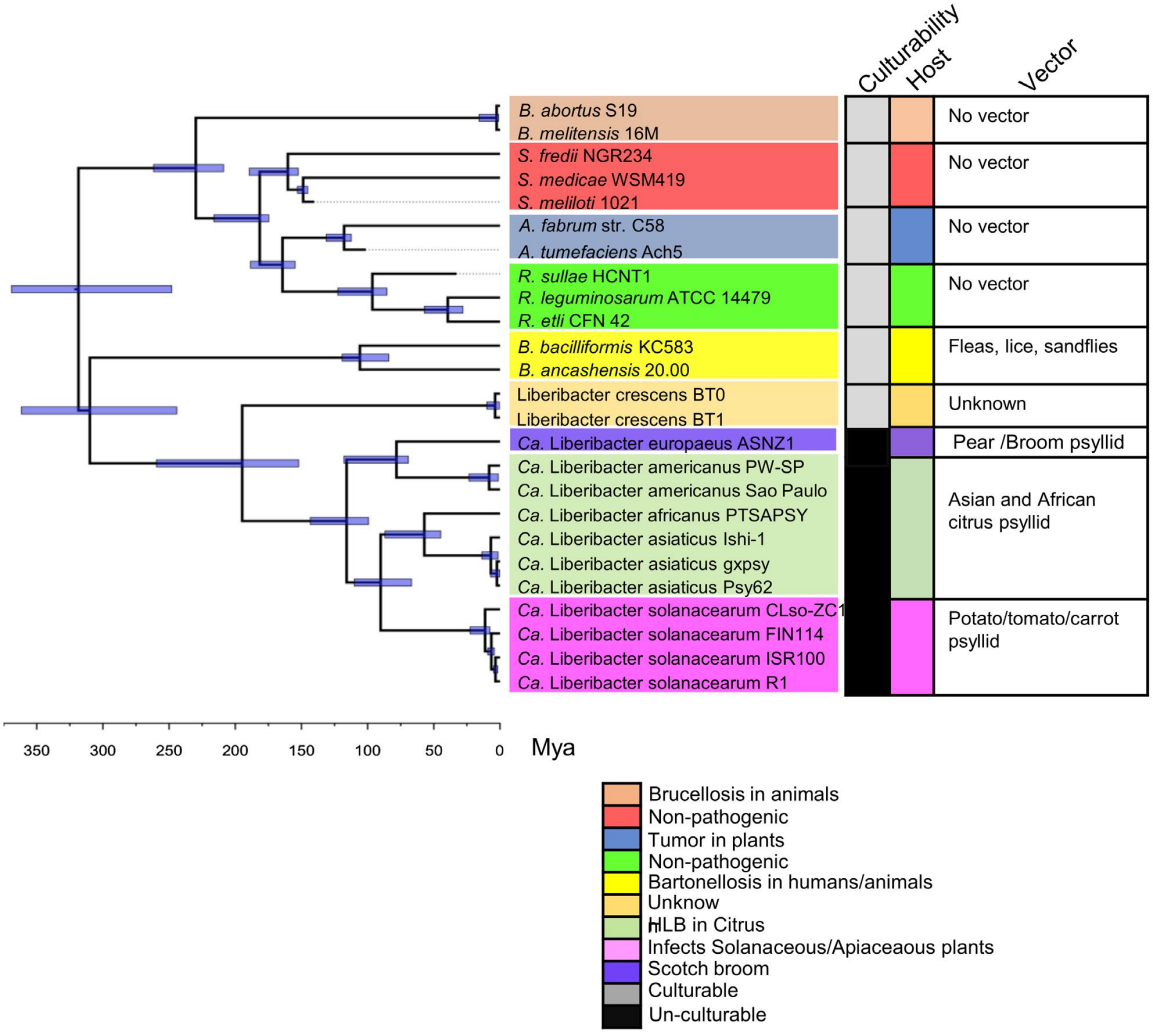
Estimated divergence times of ‘*Ca.* Liberibacter’ species and other representative *Rhizobiales* isolates. Divergence time was estimated based on a node‐calibration using MrBayes v. 3.2. The blue horizontal bars indicate the maximum (left end) and minimum (right end) ages of a specific node. The blue horizontal bars depict 95% highest posterior density of divergence times. A time scale is shown at the bottom. Mya, million years ago. The resulting phylogeny was visualized using FigTree v. 1.4.3 (Rambaut, 2012). The right panel shows culturability, host, and vector of different species

The split between ‘*Ca.* Liberibacter’ and the *Rhizobiales* was estimated to have occurred around 250–370 mya (Figure 2). Our result suggests a stepwise evolution from a common ancestor splitting first into nonpathogenic Lcr c.205 mya. Pathogenic ‘*Ca.* Liberibacter’ species then separated c.132 mya (Figure 2). The common ancestor Lam and Leu separated c.94 mya. The common ancestor of the citrus pathogens Laf and Las separated c.63 mya after they separated from the common ancestor of Lso c.100 mya (Figure 2). These results suggest evolutionary radiation of ‘*Ca.* Liberibacter’ species and given the relatively short time interval between speciation events, suggest high rates of diversification. Because Lam is basal to Las and Laf and clusters with Leu, this indicates convergent evolution of pathogenicity on citrus. It should be noted that accurate divergence time estimates in intracellular organisms are challenging due to differences in mutation rates in free‐living organisms and a lack of fossil record (Itoh *et al.*, 2002).

### 2.3 Citrus‐infecting ‘*Ca.* Liberibacter’ species have undergone significant evolution after divergence

**Figure 3:**
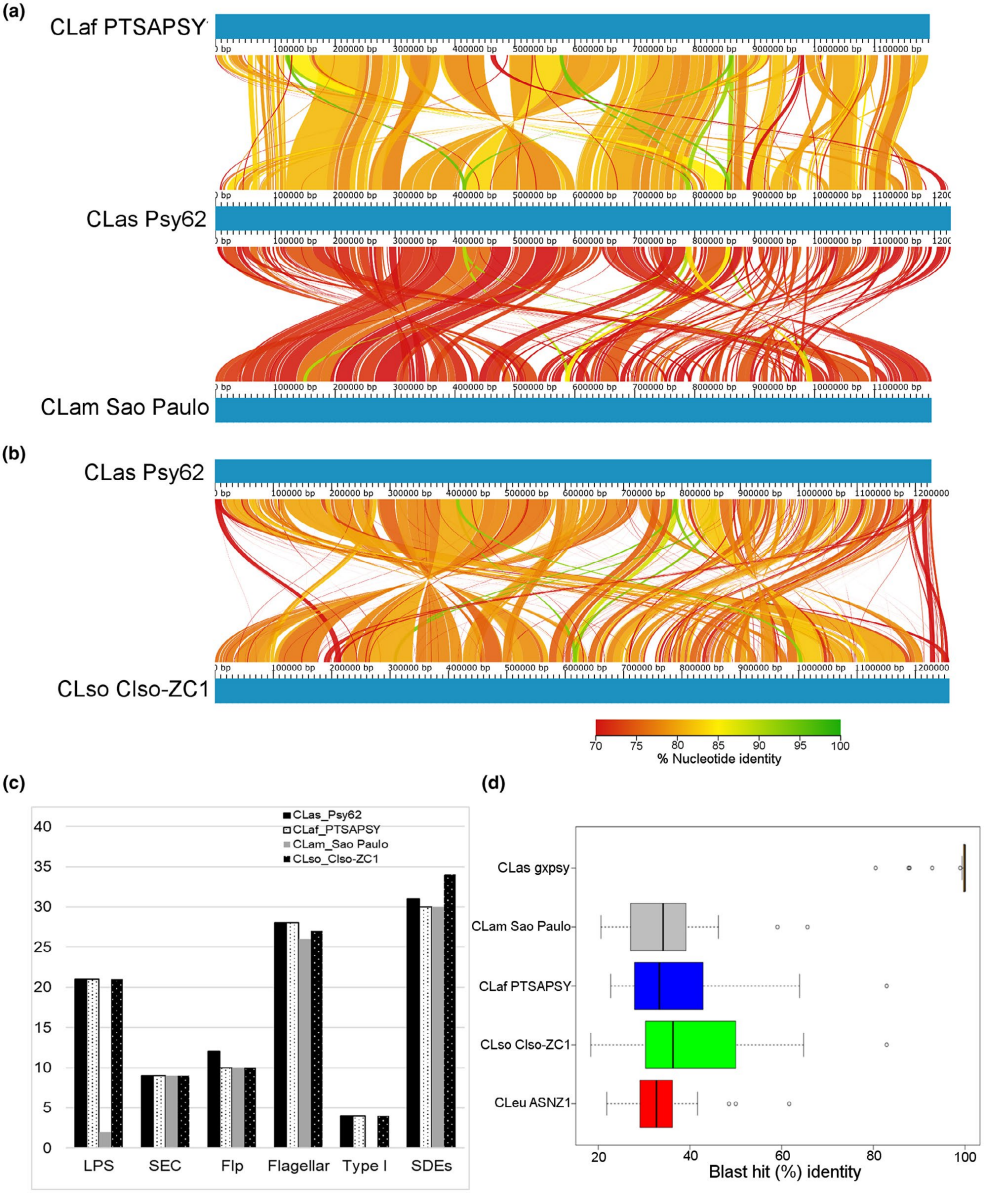
Genome‐wide comparison of pathogenic ‘*Ca.* Liberibacter’ species. Linear chromosomal maps were build using AliTV v. 1.0 visualization software, based on whole‐genome alignments with Lastz v. 1.0.4 aligner. Both panels depict pairwise comparisons, expressed as percentage of nucleotide similarity, that connect different homologous genomic regions. Genomes are completely finished and pictured in blue. (a) ‘*Ca*.Liberibacter asiaticus’ (Las) isolate Psy62, ‘*Ca*. Liberibacter africanus’ (Laf) isolate PTSAPSY, and ‘*Ca*.Liberibacter americanus’ (Lam) isolate São Paulo. (b) Las isolate Psy62 and ‘*Ca*. Liberibacter solanacearum’ (Lso) isolate Lso_ZC1. (c) Presence of different putative virulence genes in ‘*Ca.* Liberibacter’ species. LPS, lipopolysaccharide; SDE, Sec‐dependent effector; SEC, Sec translocon; Type I, Type I secretion system. (d) Amino acid similarity between *Liberibacter* SDEs. The graph indicates the percentage amino acid identity of individual effectors between the indicated strains and Las Psy62. The boxplot shows the distribution of the best BLAST hits per Las protein from each ‘*Ca.* Liberibacter’ species. ‘*Ca*. Liberibacter europaeus’, Leu

Las, Lam, and Laf species are HLB‐associated bacteria*.* While Las is distributed worldwide, Lam is mainly found in Brazil and Laf is mainly found in Africa and the neighbouring Arabian Peninsula (Bove, 2006; Gottwald, 2010). We performed pairwise genome comparisons of Las, Lam, and Laf. Consistent with the predicted speciation events, genome level comparison revealed that Las and Laf are more similar and share higher numbers of genes when compared to Lam (Figure 3). When we compared Laf and Las, we detected large regions of genome similarity in addition to several genomic rearrangements (Figure 3a). Although Las and Lam can both be found in Brazil, they exhibited fewer regions of genome similarity and extensive genomic rearrangements (Figure 3a). Lam is present in the same clade as Leu, but substantial genome rearrangement can be observed between these species (Figure S2).

Genes present only in the three citrus‐associated bacteria (Las, Lam, and Laf), but absent in other *Liberibacter* species have been included in Table S6. Las (Psy62) has 155 genes, Laf (PTSAPSY) has 92 genes, and Lam (Sao Paulo) has 43 genes that are absent in *Liberibacter* species not associated with citrus. Even though both the African and Asian psyllids and forms of the disease have been reported in the Arabian Peninsula (Bové and Garnier, 1984; Bové, 2006) and despite the observed Las and Laf genome similarity (Figure 3), only seven genes are shared by Las and Laf (Table S6). Only one gene, a type II modification methyltransferase, is shared by Las, Lam, and Laf (Table S6). Most of the genes are hypothetical proteins and annotations are unknown, so it is not possible to mine for particular categories or molecular functions required for disease in citrus (Table S6). Genes absent in all three HLB‐associated pathogens but present in only in Leu, Lcr, and Lso are included in Table S7.

We also investigated other components that may be important for ‘*Ca.* Liberibacter’ virulence. Lipopolysaccharides (LPS) are critical components of the outer membrane of gram‐negative bacteria and contribute to bacterial fitness and virulence by maintaining the structural integrity of the cell and protection from environmental stress (Raetz and Whitfield, 2002; Zhang *et al.*, 2013). LPS consist of three covalently linked domains: lipid A, a core oligosaccharide consisting of inner and outer regions, and a repeating O‐antigen (Raetz and Whitfield, 2002). While Las and Laf possess 21 genes for LPS synthesis, the majority of LPS biosynthetic loci are missing in Lam (Figure 3c), which only possesses two genes known to be involved in LPS biosynthesis (lam_190 and lam_189) (Figure 3c). In both Laf and Las, genes involved in LPS production are present primarily in three genomic clusters (Figure S3).

Flagella are responsible for rotational propulsion, promote host colonization via adherence and induce plant immunity (Rossez *et al.*, 2015). A nearly complete set of genes involved in the flagellar synthesis pathway is present in Las, Laf, and Lam (Figures 3c and S4). These flagellar syntheses genes are present in a single cluster in rhizobia, four clusters in Las, and two clusters in Laf and Lam (Figure S4). In Las one cluster is adjacent to the type I secretion system (Figure S4). Despite the presence of these clusters, flagellated Las has not been observed in planta (Bove, 2006). FlaA, the major component of the flagellar filament in rhizobia is present, whereas FlaB, FlaC, and FlaD, minor components of the flagellar filament, are missing in ‘*Ca*. Liberibacter’ species (Tambalo *et al.*, 2010).

The ability to manipulate host and vector is necessary to promote ‘*Ca.* Liberibacter’ survival and disease development. While ‘*Ca.* Liberibacter’ lacks the type III secretion system, all three citrus‐infecting species possess genes that should enable the formation of the general secretory pathway (Sec‐translocon), which is one of the chief routes for bacterial protein translocation from the cytoplasm (Figure 3c). SDEs are potential virulence factors (Segers *et al.*, 2011). A total of 31 SDEs were predicted in Las genomes, of which 27 are conserved across 24 isolates. Lam and Laf both have 30 SDEs. Interestingly, only one SDEs is shared among these three ‘*Ca*. Liberibacter’ species with an amino acid sequence identity of 60%–80% (Figure 3d). Lam and Leu share five SDEs with 60%–68% amino acid identity (LAM_RS02165, LAM_RS00350, LAM_RS04385, LAM_RS05125, and LAM_RS03975).

The type I secretion machinery consists of three proteins localized in the cell envelope: TolC (an outer membrane export protein), HlyD (a membrane fusion protein, which links inner and outer membrane proteins), and PrtD (an ABC transporter) (Splitz *et al.*, 2018). The Las and Laf type I secretion system consists of TolC, HlyD, and PrtD. However, Lam has lost the type I secretion system after divergence (Figure 3c). Lam is most closely related to Leu, which has also lost the type I secretion system and cannot produce LPS (Figure S4b). The functionality and contribution of the type I secretion system and their substrates to the virulence of ‘*Ca*. Liberibacter’ species remain to be determined.

### 2.4 Las is an emerging pathogen distinct from other *Liberibacter* species

Lso is a phloem‐limited emerging pathogen that infects apiaceous and solanaceous crops. We performed pairwise genome comparisons of Lso haplotype B, which infects tomato and potato and citrus‐infecting Las. Lso ZC1 and Las Psy62 share high genome similarity (Figure 3b). Interestingly, Lso is more similar to Las than Las to Lam, which is also HLB‐associated (Figure 3a,b). Despite regions of genome synteny, multiple genome rearrangements can be observed between Lso and Las (Figure 3b). Lso is predicted to have separated c.100 mya from the common ancestors Las and Lam (Figure 2)*.* Whole‐genome comparisons suggest several recombination events have occurred since the divergence of these two species (Figure 3a,b).

We also compared the presence of potential virulence components in Lso and Las. Lso has homologs of all the LPS genes present in Las and a nearly complete set of genes involved in the flagellar synthesis pathway in two different genomic clusters (Figures 3c and S4). Similarly, Lso also contains all the genes that should enable the formation of the Sec‐translocon and the three type I secretion components (Figure 3c). However, 34 SDEs were identified in Lso, with only seven proteins showing >43%–60% sequence similarity to Las SDEs (Figure 3d).

### 2.5 Horizontal gene transfer in ‘*Ca*. Liberibacter’ species

We identified several genes in ‘*Ca*. Liberibacter’ species that were acquired through horizontal gene transfer (HGT) (Table S3). HGT candidate genes were identified by BLASTP searches against the NCBI‐nr database, using deduced amino acid sequences of ‘*Ca*. Liberibacter’ species as a query. Loci absent from other members of the *Rhizobiacea* were classified as acquired through HGT. There are four Las HGT clusters, some of which are also present in other ‘*Ca.* Liberibacter’ species.

The predicted function of HGT loci was diverse, including a type II restriction endonuclease, transporters, and the mevalonate pathway. The mevalonate pathway is an important metabolic pathway that provides cells with bioactive molecules essential for multiple cellular processes and virulence (Heuston *et al.*, 2012). Considering the importance of the mevalonate pathway for metabolism, these genes could be attractive antibiotic targets. The iron transport and assimilation (ITA) gene cluster was only present in Lso and may be important for host adaptation (Lin *et al.*, 2011). Genes present in the ITA cluster share homology with the genes present in species within *Proteus* and *Providencia* genera that are pathogenic on humans and animals.

**Figure 4:**
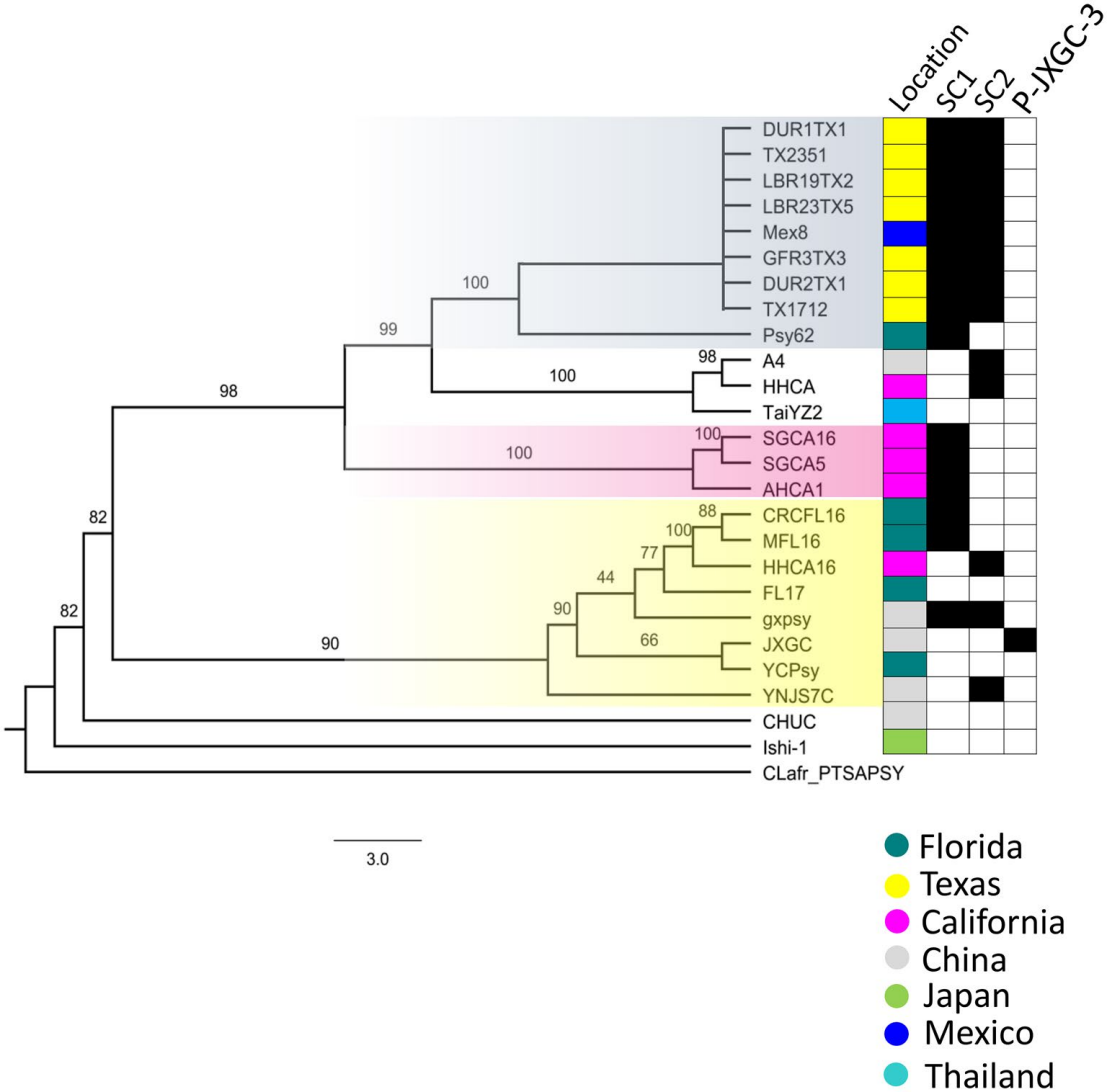
Phylogenetic analysis of ‘*Ca.* Liberibacter asiaticus’ (Las) isolates. Maximum‐likelihood phylogeny of Las based on core orthologous genes. In total, 765 orthologous genes were concatenated, and a maximum‐likelihood approach was used to generate the phylogeny with 1,000 bootstrap replicates. ‘*Ca.* Liberibacter africanus’ isolate PTSAPSY was used as the outgroup. Bootstrap values are indicated at each node. On the right are the locations where each isolate was collected and the prophages present in these isolates. The resulting phylogeny was visualized using FigTree v. 1.4.3 (Rambaut, 2012)

Genes related to virulence, antibacterial colicin protein, and a restriction‐modification (R‐M) system are present in prophages (Jain, *et al.*, 2015; Zheng *et al.*, 2018). Prophages have been suggested to assist in suppressing plant defences and activation of prophage may limit host range and culturability (Fleites *et al.*, 2014; Jain, *et al.*, 2015). The Las genome has three complete chromosomal prophages: Type 1 (SC1), Type 2 (SC2), and Type 3 (P‐JXGC‐3) (Figure S5) (Zhang *et al.*, 2011; Zheng *et al.*, 2018). More than 50% of the genes are highly similar (>85% amino acid identity) between SC1, SC2, and P‐JXGC‐3 (Figure S5). The presence of these three prophages is variable among isolates from different locations (Figure 4). Chromosomal prophages are the most variable horizontally transferred genes in ‘*Ca.* Liberibacter’ (Figure S6). The Lam genome has two prophages, SP1 and SP2, while Laf has one prophage (Wulff *et al.*, 2014; Figure S6). The Leu and Lso genomes both contain two prophages (Figure S6). In addition to these prophages, there is a remnant of a Type 4 prophage in Las that is also present in Lam, Laf, and Leu (Dominguez‐Mirazo *et al*., 2019) (Figure S5b). The Type 4 prophage differs in Las isolates with the presence or absence of other phages (Figure S5b). The importance of these prophages for bacterial virulence and host range remains to be addressed.

### 2.6 Las phylogeny indicates different sources of introduction into citrus‐producing areas

In the United States, Las was first detected in Florida in 2005 (Gottwald, 2010). In Texas, Las was first detected in 2012 (da Graça *et al*., 2015). In California, Las was first detected in 2012 from a single urban citrus tree in the city of Hacienda Heights (Kumagai *et al.*, 2013). Three years later, multiple Las isolates were found in the city of San Gabriel, California, which is 25 km from Hacienda Heights (Yan *et al.*, 2016; Dai *et al.*, 2019). Since then, Las has been detected in psyllids or citrus trees in multiple urban locations in southern California with the number of positive trees reaching more than 1,700 and the number of infected trees increasing on a daily basis (https://citrusinsider.org/). In order to investigate the genetic diversity of Las and track the source of introduction, we analysed Las genomes that were isolated from China, Japan, Florida, Texas, Mexicali, and California. To facilitate a high‐resolution comparison of sequenced Las isolates, core orthologous genes were identified. The core genome common to all 11 isolates sequenced in this study as well as 15 isolates from the NCBI database was determined. When comparing *Liberibacter* isolates with each other, we were able to identify a larger number of orthologous genes (765). The 765 identified core orthologous genes were concatenated and a maximum‐likelihood approach was employed to investigate phylogeny. The phylogenetic analysis using core orthologous sequences refined the genetic relatedness between the Las isolates.

Different clusters of Las were observed, including three branches of Californian isolates (Figure 4). Among the Californian isolates, the San Gabriel (SGCA16 and SGCA5) and Anaheim (AHCA1) isolates cluster in a single separate clade. In contrast, the two Hacienda Heights isolates (HHCA and HHCA16) are not present on the same clade and do not cluster with isolates collected from the neighbouring cities of San Gabriel or Anaheim (Figure 4). The HHCA16 is clustered in a clade with isolates from Florida and China (Figure 4). The HHCA Hacienda Heights isolate, which was isolated from the first detected Las‐positive citrus tree in California, is present in a clade containing the Chinese isolate A4 and Thai isolate TaiYZ2 (Figure 4). The Texan isolates form a single clade closely related to Florida Psy62. Psy62 is the first sequenced Las isolate that was obtained from a psyllid in Florida (Duan *et al.*, 2009). In addition, the isolate collected in Mexicali, Mexico (Mex8) clusters with Texan isolates, despite the close proximity of Mexicali to California.

These findings indicate that Florida may have been the source of the introduction of Las to Mexico and Texas. The phylogeny is also consistent with multiple Las introductions in California. The inclusion of additional genomes from different geographical locations will bring further insights into the dissemination patterns of HLB.

### 2.7 SDEs are highly conserved in Las genomes and differentially expressed in host and vector

**Figure 5:**
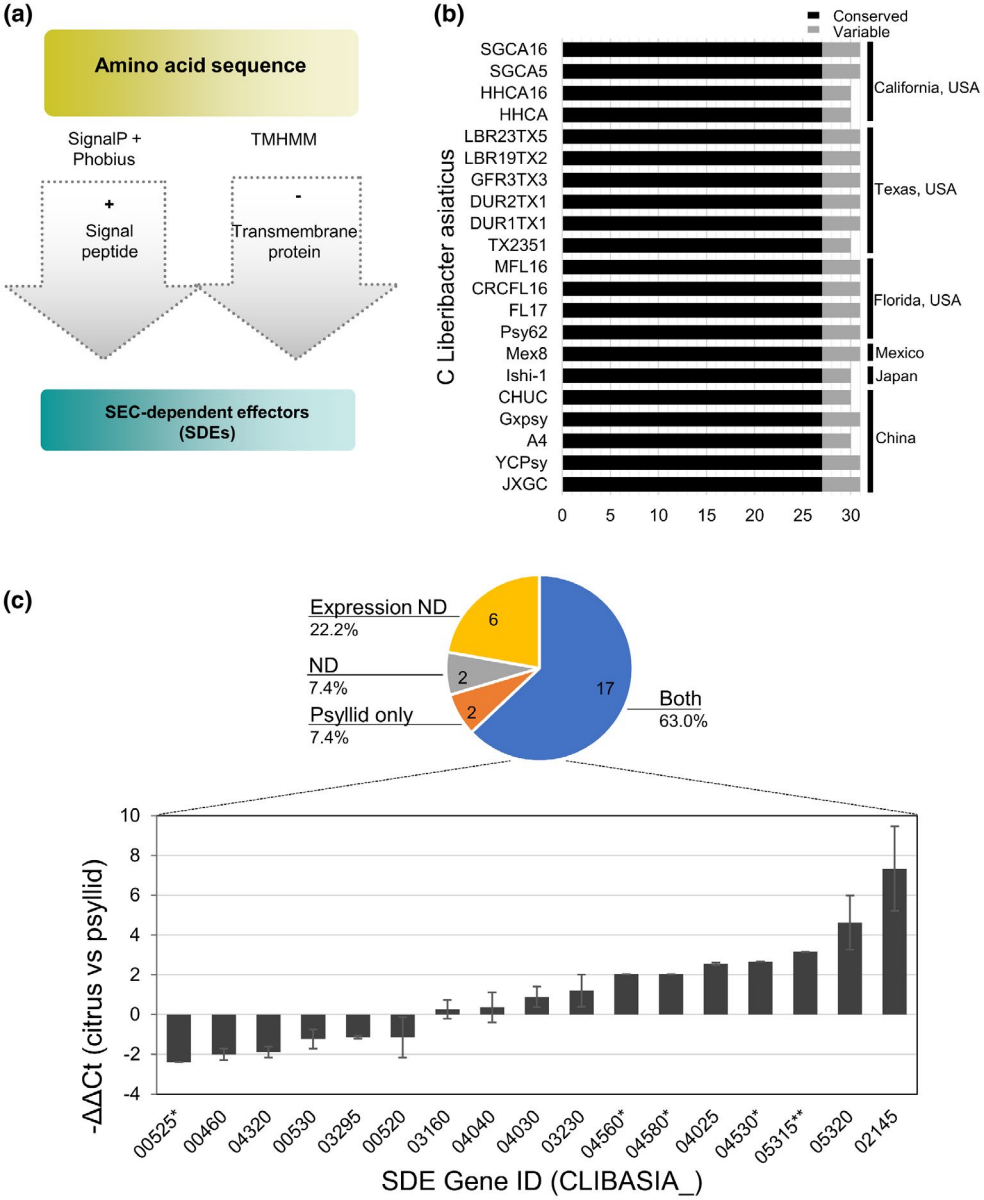
SEC‐dependent effectors present in ‘*Ca.* Liberibacter asiaticus’ (Las) isolates. (a) Flow chart showing prediction of SEC‐dependent effectors (SDEs) in Las isolates. SDEs were identified based on the presence of a signal peptide in SignalP v. 3, SignalP v. 4.1, and Phobius v. 1.01. Proteins with predicted transmembrane domains identified by TMHMM were removed. Next, proteins unlikely to be freely secreted were manually removed. (b) Conserved and variable SDEs present in different Las isolates from different locations. (c) Differential expression for Las core SDE genes between the Valencia citrus (*Citrus sinensis*) and psyllid (*Diaphorina citri*), determined by quantitative reverse transcription PCR (RT‐qPCR). The 27 core SDE genes were categorized according their expression pattern in the pie chart. Two SDEs are only expressed in psyllids (the orange section) and 17 are expressed in both hosts (the blue section). Six predicted SDEs could be amplified from genomic DNA of Las‐infected samples but did not show significant differences in the *C*_t_ values from RT‐qPCR using RNA extracts comparing with the noninoculated controls, therefore they are classified as “Nondetectable expression” (Expression ND, the yellow section). Two SDEs could not be examined because we were unable to design primers to amplify the gene and they are classified as “Nondetectable” (ND, the green section). The relative expression for the 17 *SDE* genes in citrus versus psyllids is shown in the bar chart, indicating −ΔΔ*C*_t_ values using *16S rRNA* abundance as the internal control. Positive values indicate greater expression levels in citrus and negative values indicate greater expression levels in psyllids. Error bars indicate standard deviation between two independent experiments. *Data obtained from Prasad *et al*. (2016); **Data obtained from Pagliaccia *et al*. (2017)

Effectors are collectively required for virulence in different pathogens (Dou and Zhou, 2012; Toruno *et al.*, 2016). Effectors are secreted proteins that leave the pathogen cell and are capable of modifying their host (Toruno *et al.*, 2016). As discussed above, Las possesses type I secretion system and SDEs. We sought to identify a stringent list of SDE effectors that are potential virulence proteins in Las. In order to identify core SDEs across Las, clustering analysis was performed based on sequence identity using CD‐HIT software with an identity threshold set to 95% (Li and Godzik, 2006). Homologous proteins shared a minimum of 95% amino acid sequence identity. Proteins were manually filtered to remove those unlikely to be released from the bacterial cell. A total of 31 SDEs proteins were predicted in Las genomes, of which 27 are core effectors (Figure 5). The core SDEs that were missing in one or more isolates were confirmed by PCR and sequencing. If the SDEs were absent from the isolates, not available for PCR, they were not included in the core list. Of the 27 core SDEs in Las, six are also found in Lso strain Clso‐ZC1 (CLIBASIA_02145, _02075, _04580, _03120, _03160, and 00,460).

Given the importance of SDEs during host–pathogen interactions, we sought to determine the expression pattern of the core SDEs in two organisms: citrus and psyllids. A one‐step reverse transcription‐quantitative PCR (RT‐qPCR) protocol was employed to determine the transcript levels of each individual *SDE* using gene‐specific primers. We examined samples from two citrus types: Valencia sweet orange (*Citrus sinensis*) and alemow (*Citrus macrophylla*), from Florida. The expression of SDEs showed similar patterns in these two citrus species (Figures 5c and S7).

The SDE expression profile displayed a wide range of expression levels for each gene: from nondetectable, only expressed in one of the organisms, to expressed in both citrus and psyllids (Figure 5c). In the last case, relative expression levels were determined. Six core SDEs (CLIBASIA_00185, _04055, _04405, _04410, _04690, and _05330) were considered “not detectable” for expression in either citrus or psyllids because the *C*_t_ values between noninfected controls and Las‐infected tissues showed no significant differences (Figure 5c). To this end, we could not exclude the possibility that these SDEs were still expressed but at a level below the limit of detection in our assay. It is also possible that they are expressed at a different stage of host and/or vector colonization than in tissues with symptoms from which our samples were collected. Nonetheless, reliable conclusions could not be made based on the current data. Two predicted core SDEs (CLIBASIA_01000 and CLIBASIA_04970) did not amplify by PCR even using total DNA extracts as the templates. Despite several trials, we were unable to design PCR primers that allow the examination of these two genes.

Our current data revealed the expression profiles for 19 core SDEs in citrus versus psyllids. Among them, CLIBASIA_00460, _00525, and _00530 seemed to have higher expression in psyllids, while CLIBASIA_02075 and CLIBASIA_03120 were found expressed only in psyllids. A set of six genes (CLIBASIA_00520, _03160, _03295, _04030, _04040, and _04320) were expressed at similar levels in citrus and psyllids. The remaining eight SDEs (CLIBASIA_02145, _03230, _04025, _04560, _04580, _04530, _05315, and _05320) were all expressed at higher levels in citrus than in psyllids (Figure 5c). These results support a role for SDEs during Las interactions with both citrus and psyllids. The different expression levels among SDEs also indicate diversity of biological functions in the interactions between the bacterium, the insect vector, and the plant hosts.

## 3 DISCUSSION

HLB is a longstanding disease observed by growers in the Chaoshan area of Guangdong, China dating back to the late 1880s (Lin, 1956). Three ‘*Ca.* Liberibacter’ species, Las, Lam, and Laf, have been found to associate with HLB (Bove, 2006), with Las being the most widespread and the most damaging (Gottwald *et al.*, 2007; Hodges *et al.*, 2015). Lso is also an important emerging disease of solanaceous and apiaceous plants. In this study, we employed a comparative genomics approach to shed light on the evolution, relatedness, and potential virulence components of ‘*Ca.* Liberibacter’ species.

Evolutionary analyses revealed that different ‘*Ca.* Liberibacter’ species underwent a stepwise divergence toward pathogenicity. The split between ‘*Ca.* Liberibacter’ species and the *Rhizobiales* is estimated to be 250–370 mya, followed by stepwise evolution and splitting into nonpathogenic and pathogenic species. In our current phylogenetic analyses of *Liberibacter*, we have used the time split of *Sinorhizobium*/*Agrobacterium* to calibrate the molecular clocks (Chriki‐Adeeb and Chriki, 2016). It is important to note divergence time calculations have several limitations, including the uncertainty of the split between *Sinorhizobium* and *Agrobacterium* as well as differences in mutation rates in free‐living organisms compared to host‐dependent intracellular organisms (Itoh *et al.*, 2002; Chriki‐Adeeb and Chriki, 2016). Despite these limitations, divergence time estimates can still provide a framework to understand the evolution of pathogenic *Liberibacter*. After divergence, extensive gene loss occurred in ‘*Ca.* Liberibacter’ species, thus resulting in reduced metabolic capability and reduced capabilities for DNA repair (Duan, *et al.*, 2009). ‘*Ca.* Liberibacter’ species possess a reduced genome size of c.1.2 Mb (Lcr is free‐living with genome size of 1.5 Mb), while free‐living members of the *Rhizobiaceae*, such as *Agrobacterium* and *Sinorhizobium*, have much larger genome sizes (2.8–7.3 Mb) (Capela *et al.*, 2001; Leonard *et al.*, 2012; Huang *et al.*, 2015). Lai *et al. *(2016) have identified loci in Lcr that are absent in Las. However, the majority of the Lcr loci do not have a known or predicted function. ‘*Ca.* Liberibacter’ is adapted to the niche of an intracellular environment, relying on external nutrients and metabolites. For example, Las is unable to synthesize multiple amino acids from metabolic intermediates and relies on the host and vector to import these amino acids (Duan *et al.*, 2009). The Las genome contains 40 ABC transporters for transport of proteins, which is considerably higher than other intracellular bacteria of similar genome size (Duan *et al.*, 2009). There were surprisingly few genes in common between Las, Laf, and Lam that were not present in other *Liberibacter* species. Unfortunately, most of the genes only found in Las, Laf, and Lam are hypothetical proteins without any annotations, so it is not possible to mine for particular categories of genes or molecular functions required for disease in citrus (Table S6). An alternative hypothesis is that each *Liberibacter* species has independently evolved mechanisms to infect citrus.

Las arrived in the Americas in early 1990s and in the United States in the early 2000s (Bove, 2006; da Graca *et al*., 2016). Our phylogenetic analysis using core orthologous sequences refines the genetic relatedness between Las isolates, demonstrating that different isolates are present in Florida, Texas, and California. Florida isolates primarily cluster with those from Asia and exhibit greater diversity, consistent with the early introduction of Las and multiple introduction events. In California, Las was first detected in 2012 in a backyard citrus with multiple grafts in the city of Hacienda Heights (Kumagai *et al.*, 2013). In 2015, multiple Las‐infected trees were found in San Gabriel (Yan *et al.*, 2016). Since then, Las has been detected in over 27 cities in southern California (Dai *et al.*, 2019). All Las‐positive citrus trees identified to date in California are from backyards, not commercial citrus groves. San Gabriel and Anaheim isolates are present in the same clade. However, the two sequenced Hacienda Heights isolates (HHCA and HHCA16) possess unique phylogeny. Consistent with the phylogenetic differences, Californian strains can also harbour different prophages (Zheng *et al.*, 2017; Dai *et al.*, 2019). Our study supports previous hypotheses for multiple Las introductions in California and indicates that there were at least three separate introduction events, highlighting the danger of unauthorized and unregulated moving and/or importing of citrus into an area (Gergerich *et al.*, 2015; Zheng *et al.*, 2017; Dai *et al.*, 2019). However, inclusion of more complete Las genomes will provide clear insights into the source of Las introduction in different U.S. states. It is also currently unknown if these phylogenetic differences are predictive of differences in virulence between distinct Las isolates.

Las, Laf, and Lam cause similar HLB symptoms despite clear genetic differences. We identified differences in the ability to produce LPS and type I secretion between Las, Laf, and Lam. Mutations in genes required for LPS biosynthesis can severely affect bacterial survival in planta (Kingsley *et al.*, 1993; Petrocelli *et al.*, 2012). Las and Laf have 21 genes required for LPS production. In Las, genes encoding LPS synthesis and active transport are highly expressed in planta but not in the psyllid vector (Yan *et al.*, 2013). Lam lacks the LPS gene cluster, and expression of these genes was not detected in Lam‐infected citrus (Mafra *et al.*, 2013; Wulff *et al.*, 2014). The Las and Laf genomes contain three genes encoding the type I secretion system: TolC, HlyD, and PrtD. TolC has been shown to be required for survival and pathogenicity in gram‐negative bacteria (Burse *et al.*, 2004; Reddy *et al.*, 2007). The lack of LPS and type I secretion may explain the heat sensitivity of Lam (Lopes *et al.*, 2009a, 2009b) and the absence of Lam‐positive citrus in recent years (Lopes *et al.*, 2009a).

Bacterial flagella are responsible for rotational propulsion, promote host colonization via adherence, and induce plant immunity. Flagellin has been shown to trigger immune responses in planta (Felix *et al.*, 1999). ‘*Ca.* Liberibacter’ species have most of the known flagella‐associated genes (Duan *et al.*, 2009; Zou *et al.*, 2012; Wulff *et al.*, 2014). Some flagellin genes are up‐regulated in planta and others are preferentially expressed in the psyllid (Yan *et al.*, 2013). Lam lacks FlbT, which is a flagellin activator required for flagellin expression. The lack of FlbT in Lam probably indicates that it is unable to elicit flagellin‐induced plant defence (Ferooz *et al.*, 2011; Wulff *et al.*, 2014). Although Las possesses multiple polymorphisms in the immunogenic flagellin epitope flg22, Las flg22 peptide has been reported to weakly activate plant defence in citrus (Shi *et al.*, 2018). Similarly, the immunogenic epitope of ‘*Ca.* Liberibacter’ cold shock protein is also polymorphic from recognized sequences, indicating that ‘*Ca.* Liberibacter’ may mask immunogenic molecules to avoid detection in the vector or host. Multiple tick‐borne bacterial pathogens also lack common immunogenic molecules, such as LPS, DAP‐type peptidoglycan, and flagella, presumably evading vector and host recognition (Moreno‐Garcia *et al.*, 2014).

In some intracellular bacterial pathogens, the presence of flagella confers a growth disadvantage and is energetically expensive unless it serves essential purposes such as the export of virulence factors (MacNab, 1996; Young *et al.*, 1999). Flagellated ‘*Ca*. Liberibacter’ has not been observed in planta (Bove, 2006). FlaA is present, whereas FlaB, FlaC, and FlaD are missing in ‘*Ca*. Liberibacter’ species. In rhizobia, FlaA (principal flagellin), FlaB, FlaC, and FlaD are the important components of the flagellar filament (Tambalo *et al.*, 2010). The deletion of *flaA*, *flaB*, or *flaC* genes results in a nonflagellated and nonmotile phenotype in *Agrobacterium tumefaciens* (Chesnokova *et al.*, 1997; Deakin *et al.*, 1999). Because ‘*Ca*. Liberibacter’ species lack a functional type III secretion system, it is possible that the flagellar system may be involved in the delivery of virulence components (Duan *et al.*, 2009). *Buchnera aphidicola*, a nonmotile endosymbiont pathogen, is presumed to utilize flagellar assembly genes for protein export into its host (Maezawa *et al.*, 2006).

The ability to secrete bacterial effector proteins is one of the most important virulence factors of bacterial pathogens (Dou and Zhou, 2012; Toruno *et al.*, 2016). Las possess the type I and general secretory pathway/Sec‐translocon. The essential roles of SDEs in virulence are best illustrated by phytoplasmas, which are also insect‐transmitted phloem‐colonizing bacteria (Hogenhout *et al.*, 2009). Phytoplasma SDEs are able to move through plasmodesmata and the expression of individual SDEs in *Arabidopsis thaliana* mimics disease symptoms (Hoshi *et al.*, 2009; MacLean *et al.*, 2011). SDEs of Las have been explored to understand virulence, survival, and detection (Prasad *et al.*, 2016; Pagliaccia *et al.*, 2017; Shi *et al.*, 2019). Las SDE1 targets citrus papain‐like cysteine proteases, which are induced during infection and known to be plant immune regulators (Clark *et al.*, 2018). SDE1 also induces starch formation and chlorosis after transient expression in *Nicotiana benthamiana* (Pitino *et al.*, 2018). Interestingly, few SDEs are shared between Las, Laf, and Lam. Lam and Leu, on the other hand, are more closely related and share five SDEs. Similarly, the majority of SDEs are shared between different Las isolates, indicating their importance.

In our study, we examined Sec effector expression in citrus and psyllids. Nineteen core SDEs were analysed for differential expression, revealing similar expression levels in two citrus species, but different expression patterns between vector and citrus hosts. Similar to phytoplasmas, most Las SDEs were expressed in the plant (63%) while only two (7.4%) were solely expressed in the insect vector (Hogenhout *et al.*, 2009). These results support a role for SDEs during Las colonization in citrus and HLB disease development. Differences in the magnitude and duration of Las SDE expression have also been detected (Shi *et al.*, 2019). Thus, not only does Las differentially modulate effector expression between plant host and insect vector, but effector expression is also dynamic over the course of infection (Clark *et al.*, 2018; Shi *et al.*, 2019).

Pathogenic species of ‘*Ca.* Liberibacter’ are of great importance but challenging to study because of their phloem‐limited nature, intracellular lifestyle, and inability to be cultured axenically. Our analysis of 36 *Liberibacter* genomes and other *Rhizobiales* led to a greater understanding of this important genus. We show that citrus‐infecting ‘*Ca*. Liberibacter’ species evolutionarily separated and identify potential virulence factors that could be attractive targets for disease management. Phylogenetic analysis revealed that there are distinct Las isolates in the United States and future investigations of biological and phenotypic differences between isolates should be conducted in order to inform disease management and resistance breeding programmes.

## 4 EXPERIMENTAL PROCEDURES

### 4.1 Bacterial isolates, sequencing, assembly, and annotation

The bacterial isolates used in this study and their collection sites are listed in Table S1. We sequenced the genomes of 11 Las isolates after DNA extraction from citrus plants with HLB symptoms from Florida, Texas, California, Mexico, and China (Table S1). Florida (2), Texas (5), and Mexico (1) samples were collected from commercial groves. DNA samples of the Californian isolates (2) were obtained from the California Department of Food and Agriculture and were not from commercial groves. The isolate from China (CHUC) was intercepted by the Citrus Clonal Protection Program (UC Riverside, National Clean Plant Network) in a citron (*Citrus medica*) introduction (IPPN 510) from Weishan, Yunnan, China (USDA‐APHIS‐PPQ Permit PCIP‐14‐00356) and deposited as B439 at the USDA‐ARS‐Exotic Pathogens of Citrus Collection (EPCC, Beltsville, MD, USA). Total plant DNA was extracted from infected citrus leaf midribs showing symptoms using the cetyl trimethylammonium bromide method (Sambrook, 2000). Library preparation was performed using the Illumina TruSeq DNA Nano library preparation kit (75 bp), MiSeq (300 bp) and HiSeq (150 bp). Genomic DNA was sheared with an E220 sonicator (Covaris, Inc.), followed by end repair and phosphorylation. Fragmented DNA was used to construct paired‐end libraries. Libraries were checked for size with a High Sensitivity DNA Kit (Agilent Technologies) in the Bioanalyzer 2100 (Agilent Technologies) with an expected average size of 150–250 bp. Libraries were pooled and sequenced with single‐end reads on NextSeq, paired‐end reads on MiSeq and HiSeq 4,000 systems (Illumina). Genome sequencing was performed at SeqMatic LLC (Illumina NextSeq 500, San Diego, CA, USA) and at the Genome Center at UC Davis DNA Technologies Core Facility (Ilumina). Sequenced genomes were de novo assembled with the SPAdes v. 3.10 (Bankevich *et al.*, 2012). Draft genomes were annotated with Prokka v. 1.11 and the NCBI Prokaryotic Genome Annotation Pipeline (Seemann, 2014; Tatusova *et al.*, 2016).

### 4.2 Bioinformatics analysis

ANI was calculated using pyani v. 0.2.3 (https://github.com/widdowquinn/pyani) (Pritchard *et al.*, 2016). Pairwise genome alignment was achieved by the lastz v. 1.04 program (Harris, 2007). The results were visualized using AliTV v. 1.0 (Ankenbrand *et al.*, 2017). Prophage regions were predicted using PHASTER (Arndt *et al.*, 2016).

For horizontal gene transfer (HGT), BLASTP search (*E* value <10^−5^) was performed with all putative proteins predicted in the genomes of ‘*Ca.* Liberibacter’ species in the NCBI‐nr database. Proteins that had hits with a taxonomic classification other than *Rhizobiales* were selected as candidate genes.

To predict Sec‐dependent effectors, protein sequences were screened for Sec signal peptides. Proteins possessing signal peptides for the Sec‐dependent pathway were identified using SignalP v. 3.0, SignalP v. 4.0, and Phobius v. 1.01 (Bendsten *et al*., 2004; Kall *et al.*, 2004; 2007; Petersen *et al.*, 2011). Predicted lipoproteins and transmembrane proteins were filtered from the Sec secretomes. Transmembrane topology was predicted using TMHMM v. 2.0 (Sonnhammer *et al*., 1998). SDEs predicted by at least one software package were included. Proteins were then manually filtered to remove those unlikely to be released from the bacterial cell (ABC transporters, lipoproteins, flagellar apparatus, etc.). The presence of core SDEs were confirmed by PCR and sequencing for the 11 isolates sequenced in this study (Table S2).

### 4.3 Phylogenetic analyses

Orthologous genes of Las isolates were predicted using the OrthoMCL v. 2.0 pipeline (Li *et al.*, 2003). All‐versus‐all BLASTN (*E* value <10^−5^, alignment coverage >50%) comparison of all gene sequences for each species was performed, and orthologous genes were clustered by OrthoMCL v. 2.0. The normalized scores were fed into the MCL algorithm to classify the genes into predicted orthologous gene families using a default inflation parameter of 1.5. We performed multiple alignments of gene sequences with PRANK v. 170,427 (Löytynoja, 2014). All the alignments were concatenated by FASconCAT v. 1.1, yielding a gene supermatrix (Kuck and Meusemann, 2010). A maximum‐likelihood approach was used to reconstruct the phylogenetic tree using RAxML v. 8.2 software (Stamatakis, 2006). The bootstrap was performed with 1,000 replicates. The resulting phylogeny was visualized using FigTree v. 1.4.3 (Rambaut, 2012).

### 4.4 Estimation of divergence time among ‘*Ca*. Liberibacter’ species

Based on previously published data, the divergence times of 140.10–200.87 and 100.10–149.07 mya between *Sinorhizobium* and *Agrobacterium*, respectively, were used as calibration points (Chriki‐Adeeb and Chriki, 2016). The resulting posterior distribution of divergence times were used to infer 95% posterior intervals. Ambiguously aligned regions were removed using GBlocks v. 0.91b (Castresana, 2000) with the “codon” model (−t = c), smaller block (−b4 = 3), and half gaps allowed (−b5 = a). All refined alignments were then concatenated into a supermatrix using FASconCAT v. 1.1 (Kuck and Meusemann, 2010). The Bayesian tree was inferred using MrBayes v. 3.2 (Huelsenbeck and Ronquist, 2001). Markov chain Monte Carlo (MCMC) runs were performed with one cold chain and three heated chains for 10 million generations and sampled every 1,000 generations. Chain stationarity was visualized by plotting likelihoods against the generation number using TRACER v. 1.7 (beast.bio.ed.ac.uk/Tracer). The effective sample sizes were greater than 200 for all parameters after the first 10% of generations was discarded. The resulting phylogeny was visualized using FigTree v. 1.4.3 (Rambaut, 2012).

### 4.5 Core SDE expression profiling

Core SDEs identified from Las isolates were determined for their relative expression in two organisms, citrus versus psyllids, by RT‐qPCR. Two citrus types, Valencia sweet orange (*C. sinensis*) and alemow (*C. macrophylla*), both infected with a Florida Las isolate, were included in the analysis. Midribs were freeze‐dried and ground in liquid nitrogen to a fine powder. Total RNA was extracted from 50 mg of tissue powder using the TRIzol (Invitrogen)‐based method. For RNA extraction from psyllids (*D. citri*), 10–15 adult psyllids were ground in liquid nitrogen and immediately suspended in 50 μl TRIzol. TRIzol (950 μl) was then added to the suspension to extract total RNA. RNA extracts from citrus and psyllids were treated with DNase I (Thermo‐Fisher Scientific) to remove genomic DNA. RNA concentration and quality were examined by NanoDrop spectrophotometer (Thermo Fisher Scientific) and confirmed by electrophoresis.

Expression of individual SDEs was determined by RT‐qPCR using the one‐step QuantiTect SYBR Green RT‐PCR kit (Qiagen) and the CFX96 real‐time PCR detection system (Bio‐Rad). Primers were designed based on specificity, amplicon size, and compatibility using the Primer3 0.4.0 (http://bioinfo.ut.ee/primer3-0.4.0/). Their sequences are listed in Table S3. *16S rRNA* was used as the internal reference (Yan *et al.*, 2013). Two or more independent experiments were performed for each *SDE* gene and each experiment included two technical replicates. RT‐qPCR was conducted using the following conditions: 50 °C for 30 min, 95 °C for 15 min, and then 40 cycles of 94 °C for 10 s, 58 °C for 30 s, and 72 °C for 30 s.

### 4.6 RT‐qPCR data analysis

We first determined whether *SDE* transcripts were detectable by comparing the *C*_t_ values between RNA samples from noninoculated controls and Las‐infected tissues. If the difference of the *C*_t_ values was less than 3, the gene transcripts were considered “not detectable”. For those *SDE* genes with detectable expression from both citrus and psyllids, differential expression was determined using the formula −ΔΔ*C*_t_ = −[(*C*_t Citrus CLas gene_ − *C*_t citrus CLas 16s_) − (*C*_t Psyllid CLas gene_ − *C*_t Psyllid CLas 16s_)] (Livak and Schmittgen, 2001). The *C*_t_ values used in the calculation were the average of biological replicates. At least two independent assays were performed for each gene in each host. The average and standard deviation of −∆∆*C*_t_ were calculated using values from the independent assays.

## Data Availability

The data that support the findings of this study are released in GenBank and the accession numbers are provided in Tables S1 and S2.
