# Supplementary figures and images for: Genome‐wide analyses of Liberibacter species provides insights into evolution, phylogenetic relationships, and virulence factors
Source: Mol Plant Pathol. 2020 Feb 28;21(5):716–31. doi: 10.1111/mpp.12925 (PMC7170780; doi:10.1111/mpp.12925)

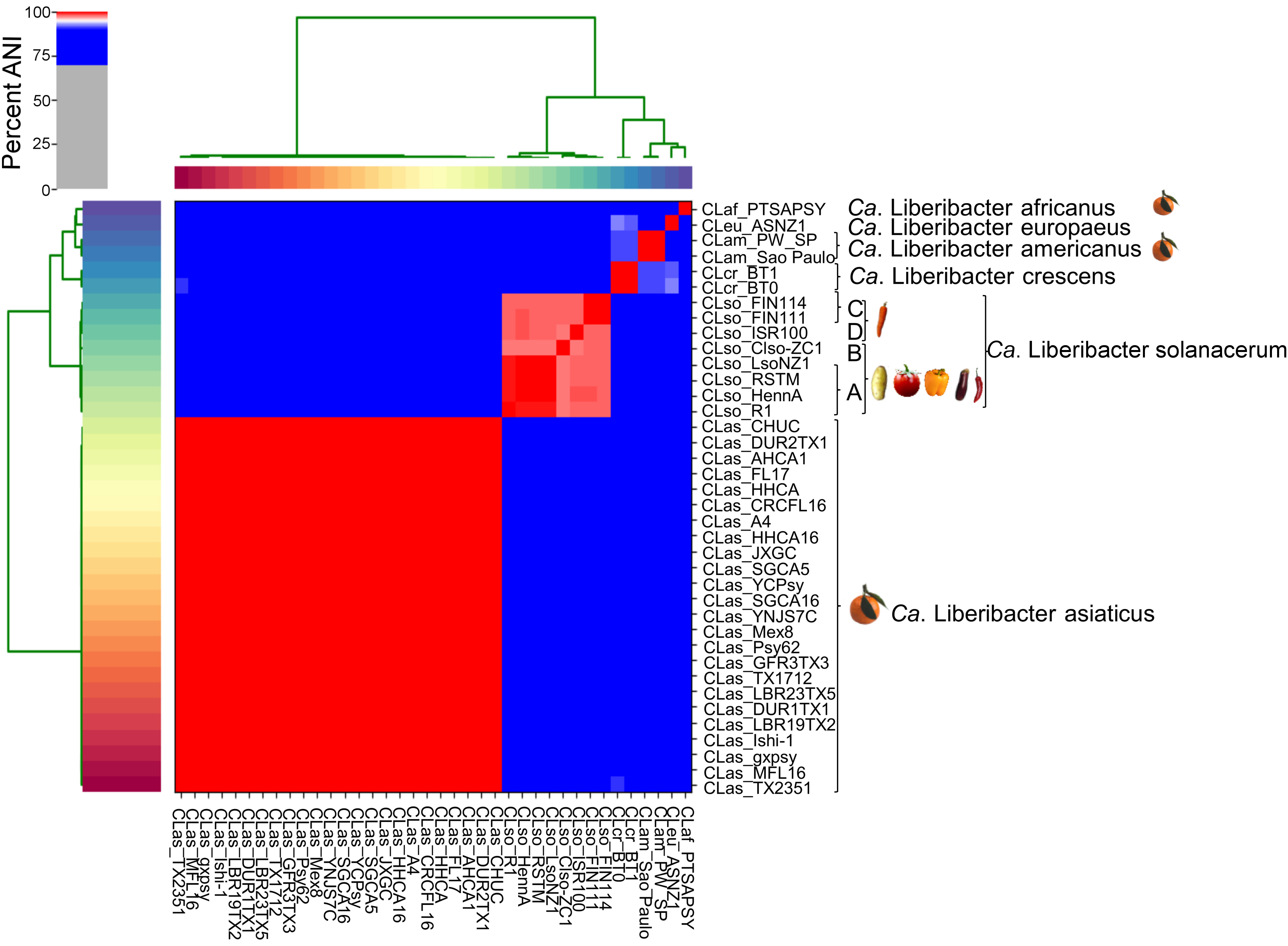

Supplement: Supplementary file 1 [file MPP-21-716-s001.tif]

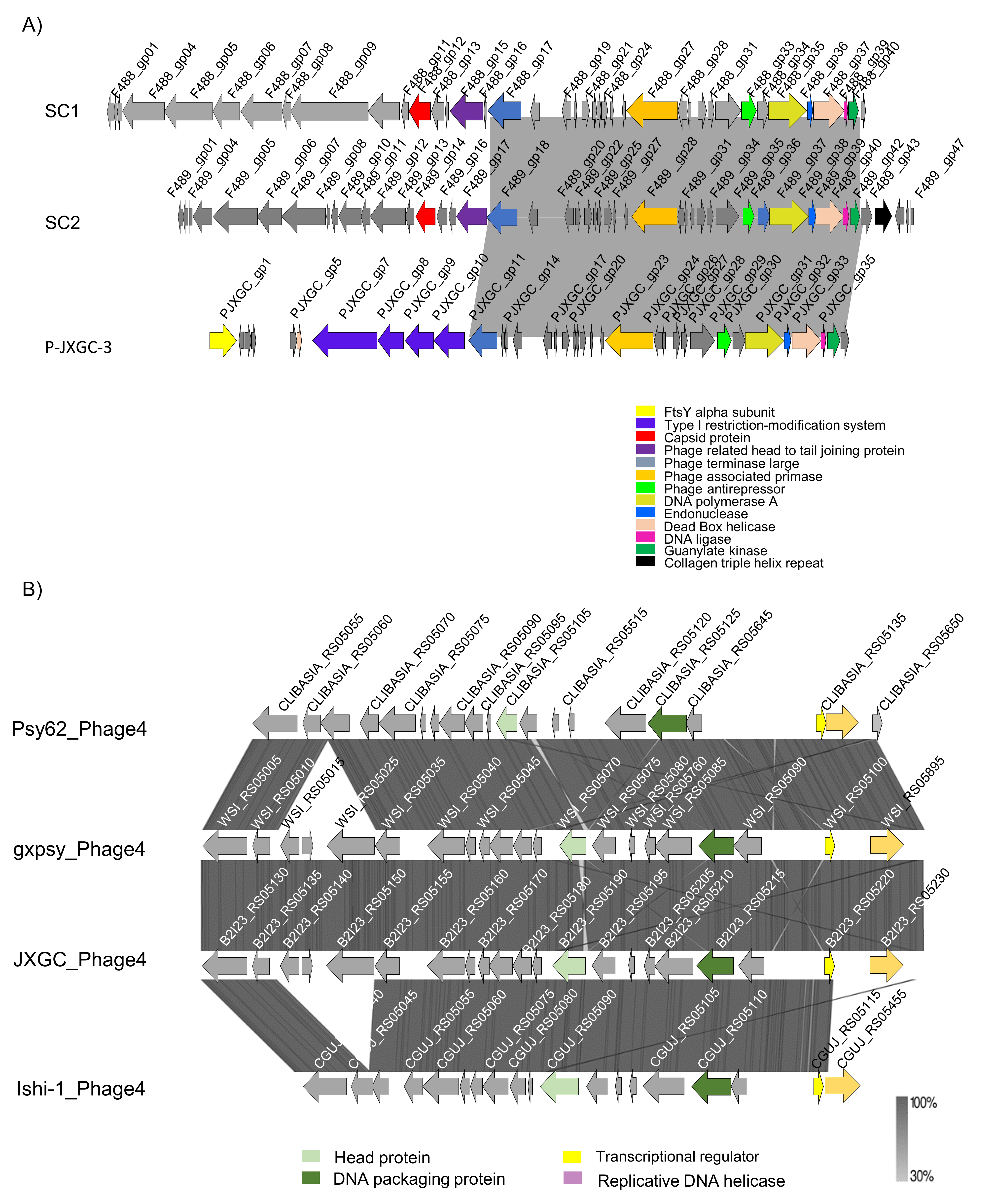

Supplement: Supplementary file 5 [file MPP-21-716-s005.tif]

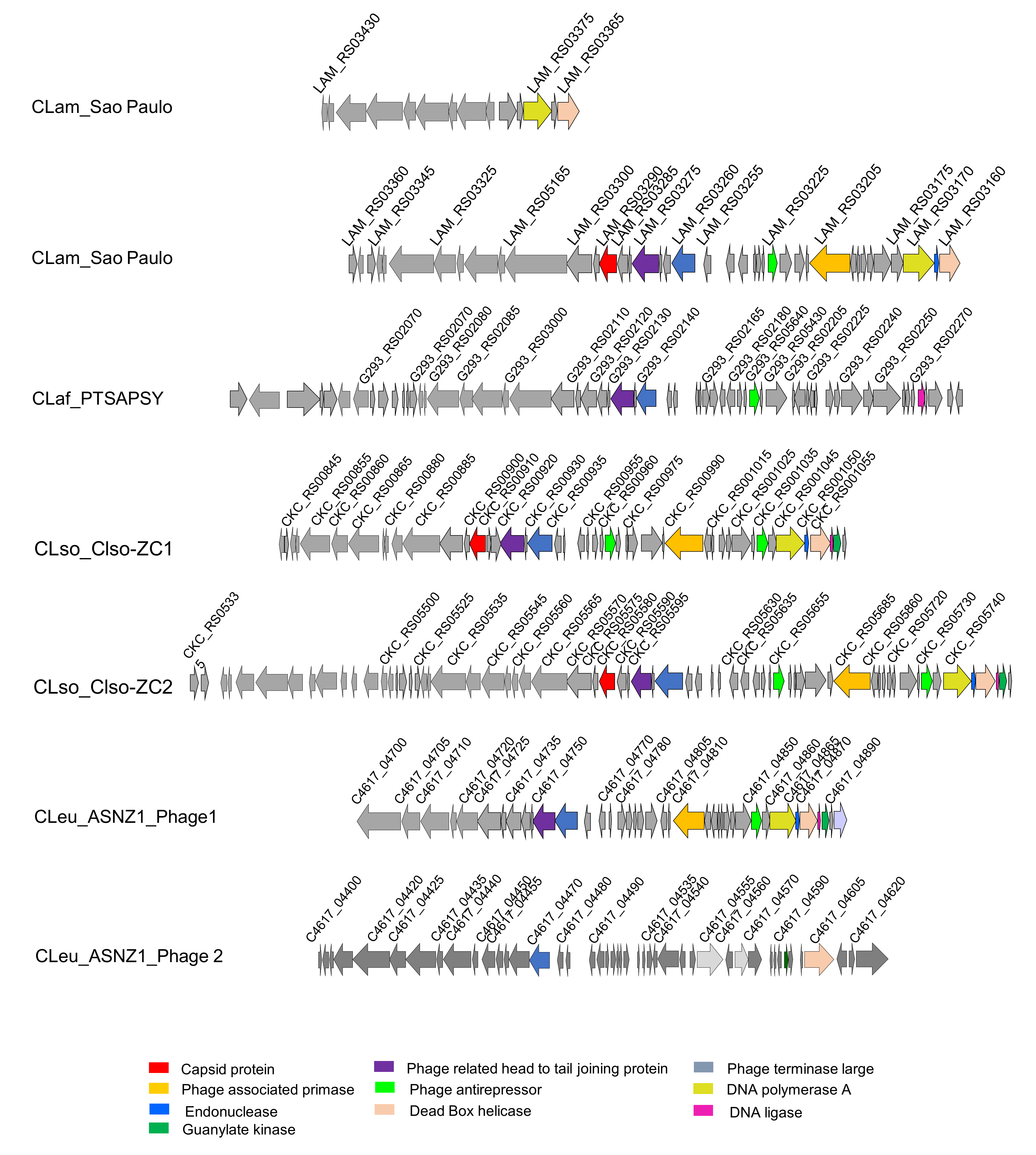

Supplement: Supplementary file 6 [file MPP-21-716-s006.tif]
