# Supplementary material for: Genome‐wide analyses of Liberibacter species provides insights into evolution, phylogenetic relationships, and virulence factors
Source: Mol Plant Pathol. 2020 Feb 28;21(5):716–31. doi: 10.1111/mpp.12925 (PMC7170780; doi:10.1111/mpp.12925)
Supplement: Supplementary file 2 [file MPP-21-716-s002.pdf]

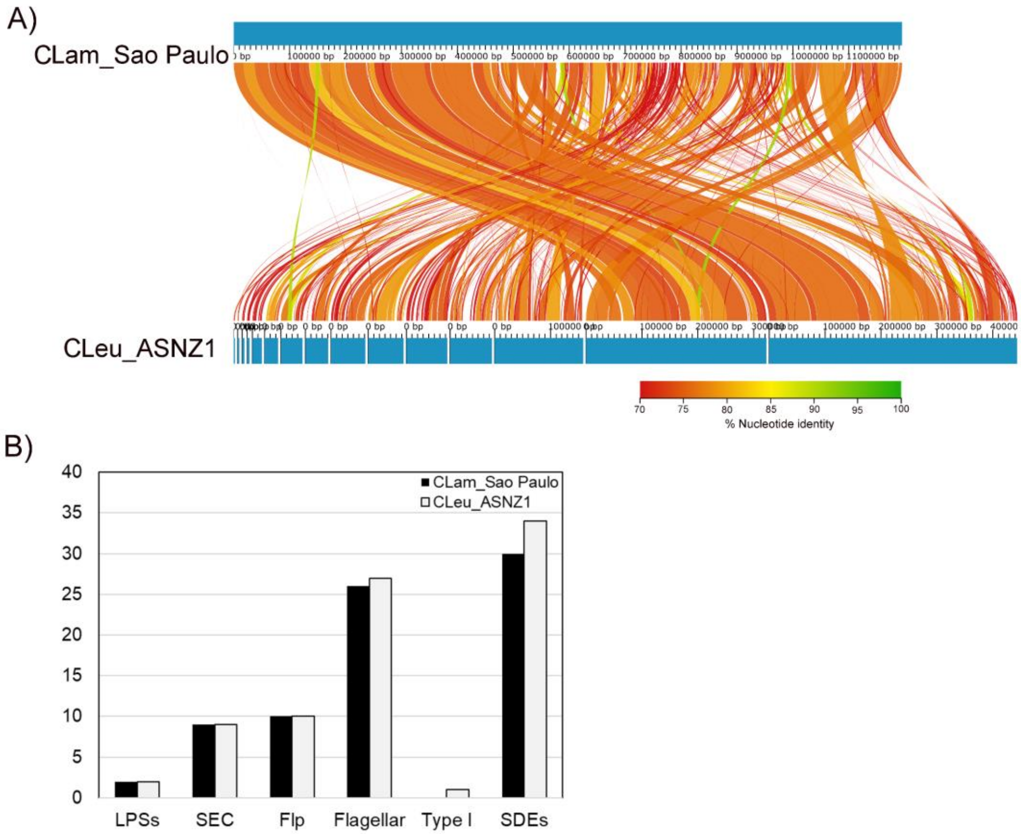

Fig. S2 Genome-wide comparison of pathogenic Lam and nonpathogenic Leu. A). Genome alignment of *C.* Liberibacter americanus (Lam, isolate Sao Paolo) and nonpathogenic *C.* Liberibacter europeaus (Leu, isolate ASNZ1). Linear chromosomal maps were build using AliTV visualization software, based on whole-genome alignments with Lastz aligner. Both panels depict pair-wise comparisons, expressed as percentage of nucleotide similarity, that connect different homologous genomic regions. Genomes are pictured in blue. Lam is a completely finished genome while Leu is not (note breaks in the blue genome depiction). **B)**. The presence of different putative virulence genes in *C.* Liberibacter species. LPS = Lipopolysaccharide, SDE = Sec-dependent effectors, SEC = Sec Translocon, Type I= Type I secretion system.
