# Supplementary material for: Genome‐wide analyses of Liberibacter species provides insights into evolution, phylogenetic relationships, and virulence factors
Source: Mol Plant Pathol. 2020 Feb 28;21(5):716–31. doi: 10.1111/mpp.12925 (PMC7170780; doi:10.1111/mpp.12925)
Supplement: Supplementary file 3 [file MPP-21-716-s003.pdf]

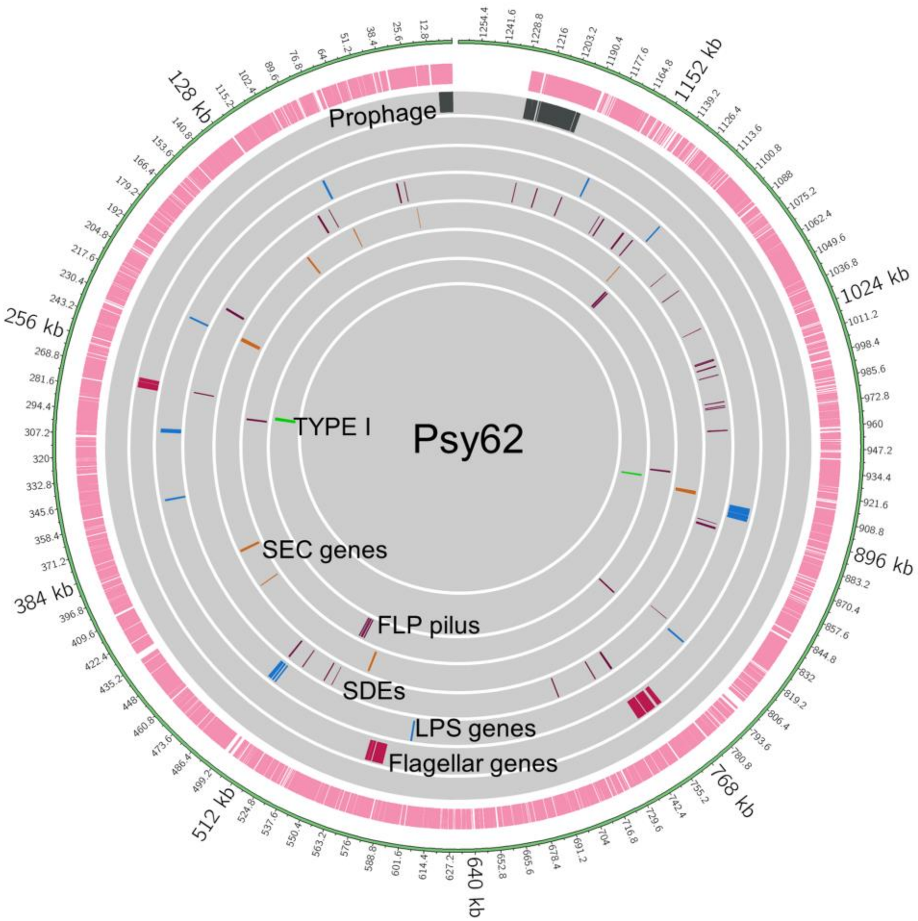

Fig. S3 Distribution of prophage and potential virulent components in the Las Psy62 genome. Circular plot depicting the genome-wide distribution of prophage and virulence components in Las Psy62. Other Las isolates harbor similar locations of the indicated genes. LPS = Lipopolysaccharide, SDE = Sec-dependent effectors, SEC = Sec translocon, Type I= Type I secretion system.
