# Supplementary material for: Genome‐wide analyses of Liberibacter species provides insights into evolution, phylogenetic relationships, and virulence factors
Source: Mol Plant Pathol. 2020 Feb 28;21(5):716–31. doi: 10.1111/mpp.12925 (PMC7170780; doi:10.1111/mpp.12925)
Supplement: Supplementary file 4 [file MPP-21-716-s004.pdf]

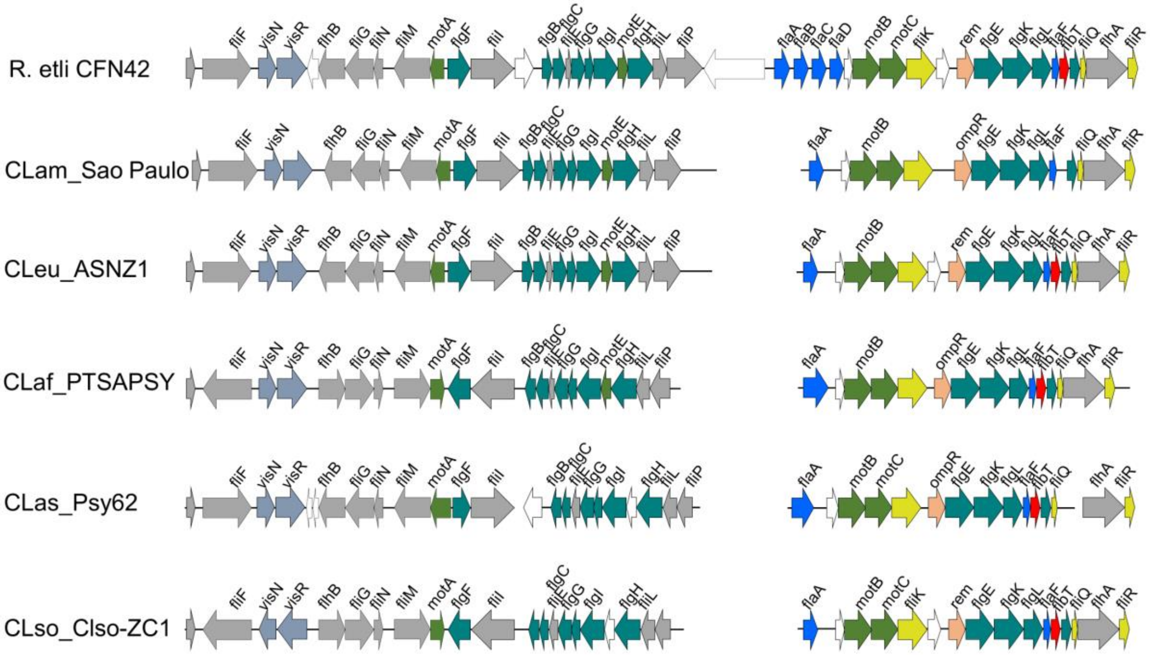

Fig. S4 Comparison of the flagellar gene cluster present in *C.* Liberibacter species and *Rhizobium etli* isolate CFN42. Genes are represented by arrows. Lam - strain Sao Paulo, Leu - strain ASNZ1, Laf - strain PTSAPSY, Las – strain Psy62, Lso - strain Clso-ZC1. Similar colors indicate similar genes. Continuous lines indicate presence in a single cluster.
