# Supplementary material for: Genome‐wide analyses of Liberibacter species provides insights into evolution, phylogenetic relationships, and virulence factors
Source: Mol Plant Pathol. 2020 Feb 28;21(5):716–31. doi: 10.1111/mpp.12925 (PMC7170780; doi:10.1111/mpp.12925)
Supplement: Supplementary file 7 [file MPP-21-716-s007.pdf]

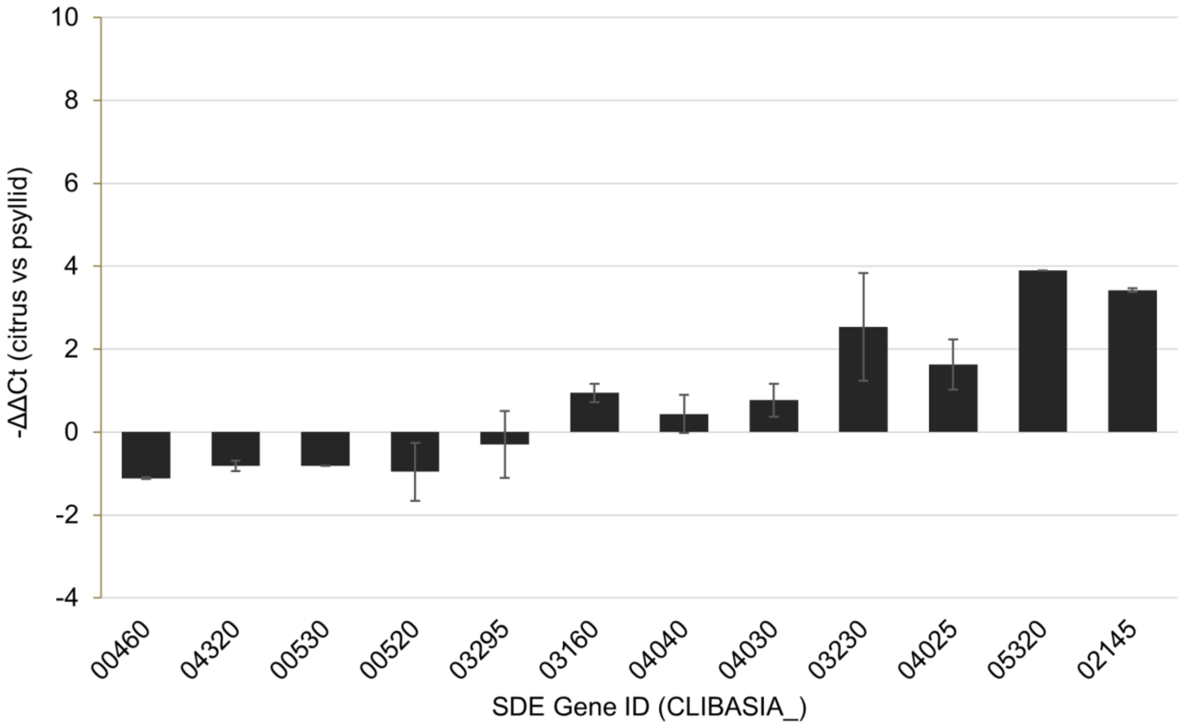

Fig. S7. SDEs present in Las isolates. Differential expression for *C*Las core SDE genes between citrus *C. macrophylla* and psyllid hosts, determined by RT-qPCR. The 27 core SDE genes were categorized according their expression pattern in the pie chart (Fig. 5C). The relative expression for the 17 *SDE* genes in citrus vs psyllids is shown in the bar chart, indicating -ΔΔCt values using *16S* rRNA abundance as the internal control. Positive values indicate greater expression levels in citrus, and negative values indicate greater expression levels in psyllids. Error bars indicate standard deviation between two independent experiments. * = data obtained from Prasad *et al.,* 2016 (79); ** = data obtained from Pagliaccia *et al.,* 2017 (Pagliaccia *et al.,* 2017).
