# Supplementary material for: Genome‐wide analyses of Liberibacter species provides insights into evolution, phylogenetic relationships, and virulence factors
Source: Mol Plant Pathol. 2020 Feb 28;21(5):716–31. doi: 10.1111/mpp.12925 (PMC7170780; doi:10.1111/mpp.12925)
Supplement: Supplementary file 8 [file MPP-21-716-s008.docx]

**Table S1. Bacterial isolates used in this study**

| Isolate ID | GenBank Accession | Species | Haplotype | DNA source | Region of isolation | Country | Collection date | Reference |
| --- | --- | --- | --- | --- | --- | --- | --- | --- |
| Las_CRCFL16 | VTLW00000000 | C. Liberibacter asiaticus | - | Plant | Lake Alfred, Florida | US | 2016 | This study |
| Las_MFL16 | VTLX00000000 | C. Liberibacter asiaticus | - | Plant | Central Florida | US | 2016 | This study |
| Las_DUR1TX1 | VTLT00000000 | C. Liberibacter asiaticus | - | Plant | Donna, Field 3, Texas | US | 2017 | This study |
| Las_DUR2TX1 | VTLS00000000 | C. Liberibacter asiaticus | - | Plant | Donna, Field 3, Texas | US |  | This study |
| Las_GFR3TX3 | VTLR00000000 | C. Liberibacter asiaticus | - | Plant | Weslaco, Field 2, Texas | US | 2017 | This study |
| Las_LBR23TX5 | VTMB00000000 | C. Liberibacter asiaticus | - | Plant | Donna, Field 1, Texas | US | 2017 | This study |
| Las_LBR19TX2 | VTMA00000000 | C. Liberibacter asiaticus | - | Plant | Donna, Field 1, Texas | US | 2017 | This study |
| Las_SGCA16 | VTLZ00000000 | C. Liberibacter asiaticus | - | Plant | San Gabriel, California | US | 2016 | This study |
| Las_HHCA16 | VTLY00000000 | C. Liberibacter asiaticus | - | Plant | Hacienda Heights, California | US | 2016 | This study |
| Las_CHUC | VTLV00000000 | C. Liberibacter asiaticus | - | Plant | - | China | - | This study |
| Las_Mex8 | VTLU00000000 | C. Liberibacter asiaticus | - | Plant | Mexicalli, Mexico | Mexico | 2018 | This study |
| Las_psy62 | NC_012985.3 | C. Liberibacter asiaticus | - | Psyllid | Florida | US | 2009 | Duan et al., 2009 |
| Las_FL17 | NZ_JWHA00000000.1 | C. Liberibacter asiaticus | - | Plant | Florida | US | 2014 | Zheng et al., 2015 |
| Las_SGCA5 | NZ_LMTO00000000.1 | C. Liberibacter asiaticus | - | Plant | San Gabriel, California | US | 2015 | Wu et al., 2015 |
| Las_AHCA1 | NZ_CP029348.1 | C. Liberibacter asiaticus | - | Psyllid | Anaheim, California | US | 2017 | Di, Z. 2018 |
| Las_HHCA | JMIL00000000 | C. Liberibacter asiaticus | - | Plant | Hacienda Heights, California | US | 2012 | Zheng et al., 2014 |
| Las_TX2351 | NZ_MTIM00000000.1 | C. Liberibacter asiaticus | - | Psyllid | Texas | US | 2017 | Kunta et al., 2017 |
| Las_TX1712 | QEWL00000000.1 | C. Liberibacter asiaticus | - | Plant | Texas | US | 2017 | Cai et al., 2018 |
| Las_YCPsy | NZ_LIIM00000000.1 | C. Liberibacter asiaticus | - | Psyllid | Guangdong | China | 2015 | Wu et al., 2015 |
| Las_gxpsy | NC_020549.1 | C. Liberibacter asiaticus | - | Psyllid | Guangxi | China | 2012 | Lin et al., 2013 |
| Las_A4 | NZ_CP010804.1 | C. Liberibacter asiaticus | - | periwinkle | Guangdong | China | 2014 | Zheng et al., 2014a |
| Las_JXGC | NZ_CP019958.1 | C. Liberibacter asiaticus | - | Plant | Jiangxi | China | 2015 | Zheng et al., 2018 |
| Las_YNJS7C | NZ_QXDO01000003.1 | C. Liberibacter asiaticus | - | Plant | Yunnan | China | 2017 | Chen et al., 2019 |
| Las_Ishi-1 | NZ_AP014595.1 | C. Liberibacter asiaticus | - | Plant and Psyllid | Okinawa | Japan | 2005 | Katoh et al., 2014 |
| Las_TaiYZ2 | CP041385.1 | C. Liberibacter asiaticus | - | Plant | Songkhla | Thailand | 2019 | Li et al., 2019 |
| Laf_PTSAPSY | NZ_CP004021.1 | C. Liberibacter africanus | - | Psyllid | Pretoria | South Africa | - | Lin et al., 2015 |
| Lam_PW_SP | NZ_AOFG00000000.1 | C. Liberibacter americanus | - | Plant | Sao Paulo | Brazil | 2011 | Lin et al. 2013 |
| Lam_Sao Paulo | NC_022793.1 | C. Liberibacter americanus | - | Plant | Sao Paulo | Brazil | - | Wuff et al., 2014 |
| Leu_ASNZ1 | PSQJ00000000.1 | C. Liberibacter europaeus | - | Psyllid | Lincoln | New Zealand | 2012 | Frampton et al., 2018 |
| Lso_LsoNZ1 | JMTK00000000.1 | C. Liberibacter solanacearum | A | Psyllid | New Zealand | New Zealand | 2010 | Thompson et al., 2015 |
| Lso_R1 | NZ_JNVH00000000.1 | C. Liberibacter solanacearum | - | Plant | California | USA | 2014 | Zheng et al., 2014b |
| Lso_HenneA | JQIG00000000 | C. Liberibacter solanacearum | A | Psyllid | Texas | USA | 2012 | Thompson et al., 2015 |
| Lso_RSTM | NZ_LLVZ00000000.1 | C. Liberibacter solanacearum | A | Psyllid | California | USA | 2015 | Wu et al., 2015 |
| Lso_Clso-ZC1 | NC_014774.1 | C. Liberibacter solanacearum | B | Psyllid | Texas | USA | - | Lin et al., 2011 |
| Lso_FIN111 | LVWB00000000 | C. Liberibacter solanacearum | C | Plant | Forssa | Finland | 2012 | Wang et al., 2017 |
| Lso_FIN114 | LWEB00000000 | C. Liberibacter solanacearum | C | Plant | Forssa | Finland | 2012 | Wang et al., 2017 |
| Lso_ISR100 | PKRU00000000 | C. Liberibacter solanacearum | D | Psyllid | - | Israel | - | Katsir et al., 2018 |
| BT-1 | CP003789 | *Liberibacter crescens* | - | Papaya | - | Puerto Rico | 1995 | Leonard et al., 2012 |
| BT-0 | CP010522 | *Liberibacter crescens* | - | Babaco pedunkle sap | - | Puerto Rico | - | Fagen,J.R. and Triplett,E.W., unpublished |
| Ach5 | CP011246-CP011249 | *Agrobacterium tumefaciens* | - | Ach5 | California | USA | - | Huang et al., 2015 |
| C58 | AE008688-AE008689 | *Agrobacterium fabrum* | - | C58 | New York | USA | - | Wood et al., 2001 |
| CFN42 | NC_007761.1 | *Rhizobium etli* | - | CFN42 | Guanajuato | Mexico | 1981 | Gonzalez et al., 2006 |
| 1021 | AL591688.1 | *Rhizobium meliloti* | - | 1021 | - | - | - | Capela et al., 2001 |
| WSM419 | CP000738 | *Sinorhizobium medicae* | - | WSM419 | Sardinia | Italy | 1981 | Reeve et al., 2010 |
| NGR234 | NC_012587.1 | *Sinorhizobium fredii* | - | NGR234 | - | Papua New Guinea | 1965 | Schmeisser et al., 2009 |
| S19 | CP000887-CP000888 | *Brucella abortus* | - | S19 | - | USA | 1923 | Crasta et al., 2008 |
| KC583 | NC_008783 | *Bartonella bacilliformis* | - | KC583 | - | - | 1909 | Hendrix et al., unpublished |
| 20 | CP010401 | *Bartonella ancashensis* | - | 20 | - | Peru | 2003 | Hang et al., 2015 |
| ATCC 14479 | CP030760 | Rhizobium leguminosarum | - | ATCC 14479 | Virginia | USA | - | Chakraborty,R. Unpublished |
| HCNT1 | NZ_PIQN01000000 | Rhizobium sullae | - | HCNT1 | Volterra | Italy | 1979 | De Diego-Diaz et al., unpublished |
| 16M | AE008917 -AE008918. | *Brucella melitensis* | - | 16M | - | - | - | DelVecchio et al., 2002 |

**REFERENCES:**

**Capela, D., Barloy-Hubler, F., Gouzy, J., Bothe, G., Ampe, F., Batut, J., Boistard, P., Becker, A., Boutry, M., Cadieu, E., Dréano, S., Gloux, S., Godrie, T., Goffeau, A., Kahn, D., Kiss, E., Lelaure, V., Masuy, D., Pohl, T., Portetelle, D., Pühler, A., Purnelle, B., Ramsperger, U., Renard, C., Thébault, P., Vandenbol, M., Weidner, S. and Galibert, F.** (2001) Analysis of the chromosome sequence of the legume symbiont Sinorhizobium meliloti strain 1021. *Proc Natl Acad Sci USA* **98**, 9877–9882

**Cai, W., Yan, Z., Rascoe, J. and Stulberg, M.J.** (2018) Draft Whole-Genome Sequence of “Candidatus Liberibacter asiaticus” Strain TX1712 from Citrus in Texas. *Genome Announc*. **6**, e00554-18.

**Crasta, O. R., O. Folkerts, Z. Fei, S. P. Mane, C. Evans, S. Martino-Catt, B. Bricker, G. Yu, L.D. and B. W. Sobral.** (2008) Genome sequence of Brucella abortus vaccine strain S19 compared to virulent strains yields candidate virulence genes. *PLoS One* **3**,e2193.

**Chen, Y., Li, T., Zheng, Z., Meirong, X. and Xiaoling, D.** (2019) Draft whole-genome sequence of a " Candidatus Liberibacter asiaticus” strain from Yunnan, China. *Microbiol Resour Announc.* **8,** e01413-18.

**DelVecchio, V.G., Kapatral, V., Redkar, R.J., Patra, G., Mujer, C., Los, T., Ivanova, N., Anderson, I., Bhattacharyya, A., Lykidis, A.,** **Reznik, G., Jablonski, L., Larsen, N., D'Souza, M., Bernal, A., Mazur, M., Goltsman, E., Selkov, E., Elzer, P.H., Hagius, S., O'Callaghan, D., Letesson, J.J., Haselkorn, R., Kyrpides, N. and Overbeek, R.** (2002) The genome sequence of the facultative intracellular pathogen Brucella melitensis. *Proc Natl Acad Sci USA*. 2002, **99,** 443-448.

**Duan, Y.P., Zhou, L.J., Hall, D.G., Li, W.B., Doddapaneni, H., Lin, H., Liu, L., Vahling, C.M., Gabriel, D.W. Williams, K.P. Dickerman, A., Sun, Y. and Gottwald T**. (2009) Complete Genome Sequence of Citrus Huanglongbing Bacterium, 'Candidatus Liberibacter asiaticus' Obtained Through Metagenomics. *Mol. Plant Microbe Interact.* **22**,1011-20.

**Frampton, R. A., Thompson, S. M., Kalamorz, F., David, C., Addison, S. M. and Smith, G. R.** (2018) Draft genome sequence of a “Candidatus Liberibacter europaeus” strain assembled from broom psyllids (Arytainilla spartiophila) from New Zealand. *Genome Announce.* **6,**e0043018.

**González V, Bustos P, Ramírez-Romero MA, Medrano-Soto A, Salgado H, Hernández-González I, Hernández-Celis JC, Quintero V, Moreno-Hagelsieb G, Girard L**, **Rodríguez, O., Flores, M., Cevallos, M.A., Collado-Vides, J., Romero, D. and Dávila, G.** (2003) The mosaic structure of the symbiotic plasmid of Rhizobium etli CFN42 and its relation to other symbiotic genome compartments. *Genome Biol***. 4,**R36

**Hang, J., Mullins, K.E., Clifford, R.J., Onmus-Leone, F., Yang, Y., Jiang, J., Leguia, M., Kasper, M.R., Maguiña, C., Lesho, E.P., Jarman, R.G., Richards, A.L. and Blazes, D.** (2015) Complete genome sequence of Bartonella ancashensis strain 20.00, isolated from the blood of a patient with verruga peruana. Genome Announc. **3,**3.

**Huang, Y.Y., Cho, S.T., Lo, W.S., Wang, Y.C., Lai, E.M. and Kuo, C.H.** (2015) Complete genome sequence of Agrobacterium tumefaciens Ach5. *Genome Announc.* **3,**e00570–15.

**Katoh H, Miyata S, Inoue H, Iwanami T.** (2014) Unique features of a Japanese 'Candidatus Liberibacter asiaticus' strain revealed by whole genome sequencing. *PLoS One.* **9**,e106109.

**Katsir, L., Zhepu, R., Santos, G.D., Piasezky, A., Jiang, J., Sela, N., Freilich, S. and Bahar, O.** (2018) Genome Analysis of Haplotype D of Candidatus Liberibacter Solanacearum. *Frontiers Microbiol*. **9,**2933

**Kunta, M., Zheng, Z., Wu, F., da Graca, J. V., Park, J. W., Deng, X. and Chen, J.** (2017) Draft whole-genome sequence of ‘Candidatus Liberibacter asiaticus’ strain TX2351 isolated from Asian citrus psyllids in Texas, USA. *Genome Announc.* **5**,e00170-17.

**Leonard, M.T., Fagen, J.R., Davis‐Richardson, A.G., Davis, M.J. and Triplett, E.W.** (2012) Complete genome sequence of Liberibacter crescens BT‐1. *Stand. Genom. Sci.* **7,**271–283.

**Lin, H., Han, C. S., Liu, B., Lou, B., Bai, X., Deng, C., Civerolo, E. L., and Gupta, G.** (2013) Complete genome sequence of a Chinese strain of ‘Candidatus Liberibacter asiaticus’. *Genome Announc*. **1**,e00184-13.

**Lin, H., Coletta-Filho, H.D., Han, C.S., Lou, B., Civerolo, E.L., Machado, M.A. and Gupta G.** (2013a) Draft Genome Sequence of "Candidatus Liberibacter americanus" Bacterium Associated with Citrus Huanglongbing in Brazil. *Genome Announc*. **23,**1(3).

**Lin, H., Lou, B., Glynn, J.M., Doddapaneni, H., Civerolo, E.L., Chen, C.W., Duan, Y., Zhou, L. and Vahling, C.M**. (2011) The Complete Genome Sequence of 'Candidatus Liberibacter solanacearum', the Bacterium Associated with Potato Zebra Chip Disease. *Plos One* **6**(4).

**Lin, H., Pietersen, G., Han, C., Read, D.A., Lou, B.H., Gupta, G. and Civerolo, E.L*.*** (2015) Complete Genome Sequence of "Candidatus Liberibacter africanus," a Bacterium Associated with Citrus Huanglongbing. *Microbiol. Resour. Announc.* **3**(4).

**Reeve, W., Chain, P., O'Hara, G., Ardley, J., Nandesena, K., Brau, L., Tiwari, R., Malfatti, S., Kiss, H., Lapidus, A., Copeland, A., Nolan, M., Land, M., Hauser, L., Chang, Y.J., Ivanova, N., Mavromatis, K., Markowitz, V., Kyrpides, N., Gollagher, M., Yates, R., Dilworth, M. and Howieson, J. (2010)** Complete genome sequence of the Medicago microsymbiont Ensifer ( Sinorhizobium ) medicae strain WSM419. *Stand. Genom. Sci.* **2**,77–86.

**Schmeisser C., Liesegang H., Krysciak D., Bakkou N., Le Quéré A., Wollherr A., Heinemeyer, I., Morgenstern, B., Pommerening-Röser, A., Flores, M., Palacios, R., Brenner, S., Gottschalk, G., Schmitz, R.A., Broughton, W.J., Perret, X., Strittmatter, A.W. and Streit, W.R.** (2009). Rhizobium sp. NGR234 possesses a remarkable number of secretion systems. *Appl. Environ. Microbiol.* **75**, 4035–4045.

**Li, T., Thaochan, N., Huang, J., Chen, J., Deng, X. and Zheng, Z.** (2019) Genome Sequence Resource of “Candidatus Liberibacter asiaticus” from Thailand. *Plant Dis.* https://doi.org/10.1094/PDIS-07-19-1520-A.

**Thompson, S. M., Johnson, C. P., Lu, A. Y., Frampton, R. A., Sullivan, K. L., Fiers, M. W. E. J., Ross N. Crowhurst, Pitman, A.R., Scott I.A.W., Wen A., Gudmestad, N.C., and Smith G.R.** (2015). Genomes of “Candidatus Liberibacter solanacearum” haplotype A from New Zealand and the United States suggest significant genome plasticity in the species. *Phytopathology* **105**, 863–871.

**Wang J., Haapalainen M., Schott T., Thompson S. M., Smith G. R., Nissinen A. I. and** **Pirhonen, M.** (2017) Genomic sequence of “Candidatus Liberibacter solanacearum” haplotype C and its comparison with haplotype A and B genomes. *PLoS One* **12,**e0171531.

**Wood, D.W., Setubal, J.C., Kaul, R., Monks, D.E., Kitajima, J.P., Okura, V.K., Zhou, Y., Chen, L., Wood, G.E., Almeida, N.F., Woo, L., Chen, Y., Paulsen, I.T., Eisen, J.A., Karp, P.D., Bovee, D., Chapman, P., Clendenning, J., Deatherage, G., Gillet, W., Grant, C., Kutyavin, T., Levy, R., Li, M.J., McClelland, E., Palmieri, A., Raymond, C., Rouse, G., Saenphimmachak, C., Wu, Z., Romero, P., Gordon, D., Zhang, S., Yoo, H., Tao, Y., Biddle, P., Jung, M., Krespan, W., Perry, M., Gordon-Kamm, B., Liao, L., Kim, S., Hendrick, C., Zhao, Z.Y., Dolan, M., Chumley, F., Tingey, S.V., Tomb, J.F., Gordon, M.P., Olson, M.V. and Nester EW. (**2001) The genome of the natural genetic engineer Agrobacterium tumefaciens C58. *Science* **294,**2317–2323.

**Wu, F., Deng, X., Liang, G., Cen, Y., Wallis, C., Trumble, J. T., Prager, S. and Chen, J.** (2015) De Novo genome sequence of “Candidatus Liberibacter solanacearum” from a single potato psyllid in California. *Genome Announc*. **3,**e01500-15.

**Wu, F., Kumagai, L., Liang, G., Deng, X., Zheng, Z., Keremane, M. and Chen, J.** (2015b) Draft genome sequence of ‘Candidatus Liberibacter asiaticus’ from a citrus tree in San Gabriel, California. *Genome Announc*. **3,**e01508-15.

**Wu, F., Zheng, Z., Deng, X., Cen, Y., Liang, G. and Chen, J.** (2015a). Draft genome sequence of ‘Candidatus Liberibacter asiaticus’ from Diaphorina citri in Guangdong, China. *Genome Announc*. **3**,e01316-15.

**Wulff, N.A., Zhang, S., Setubal, J.C., Almeida, N.F., Martins, E.C., Harakava, R., Kumar, D., Rangel, L.T., Foissac, X., Bove, J.M. and Gabriel, D.W*.*** (2014) The Complete Genome Sequence of 'Candidatus Liberibacter americanus', Associated with Citrus Huanglongbing. *Mol. Plant Microbe Interact.* **27**,163-76.

**Zheng, Z., Deng, X. and Chen J.** (2014a) Draft Genome Sequence of “Candidatus Liberibacter asiaticus” from California. *Genome Announc*. **2,** e00999–14.

**Zheng Z, Deng X, Chen J.** (2014b) Whole-genome sequence of “Candidatus Liberibacter asiaticus” from Guangdong, China. *Genome Announc*. **2**,e00273-14.

**Zheng, Z., Clark, N., Keremane, M., Lee, R., Wallis, C., Deng, X., and Chen, J.** (2014c) Whole-genome sequence of “Candidatus Liberibacter solanacearum” strain R1 from California. *Genome Announc*. **2,**e01353-14.

**Zheng, Z., Sun, X., Deng, X., and Chen, J.** (2015) Whole-genome sequence of ‘Candidatus Liberibacter asiaticus’ from a huanglongbing-affected citrus tree in central Florida. *Genome Announc*. **3**,e00169-15.

**Zheng, Z., Bao, M.L., Wu, F.N., Van Horn, C., Chen, J.C. and Deng, X.L.** (2018) A Type 3 Prophage of 'Candidatus Liberibacter asiaticus' Carrying a Restriction-Modification System. *Phytopathology* **108**,454-61.
