# Supplementary material for: Genome‐wide analyses of Liberibacter species provides insights into evolution, phylogenetic relationships, and virulence factors
Source: Mol Plant Pathol. 2020 Feb 28;21(5):716–31. doi: 10.1111/mpp.12925 (PMC7170780; doi:10.1111/mpp.12925)
Supplement: Supplementary file 13 [file MPP-21-716-s013.docx]

Table S6. Genes present in Huanglongbing (HLB) associated Liberibacter species, but absent in other Liberibacter isolates. HLB associated Liberibacter species include: *Candidatus* Liberibacter asiaticus (Las_Psy62), *Ca*. Liberibacter africanus (Laf_PTSAPSY), and *Ca.* Liberibacter americanus (Lam_São Paulo).

| **Las_Psy62** | **Laf_PTAPSY** | **Lam_Sao Paulo** | **Description** |
| --- | --- | --- | --- |
| CLIBASIA_00185 |  |  | hypothetical protein |
| CLIBASIA_00190 |  |  | hypothetical protein |
| CLIBASIA_00225 |  |  | hypothetical protein |
| CLIBASIA_00260 |  |  | hypothetical protein |
| CLIBASIA_00290 |  |  | hypothetical protein |
| CLIBASIA_00295 |  |  | hypothetical protein |
| CLIBASIA_00310 |  |  | hypothetical protein |
| CLIBASIA_00355 |  |  | hypothetical protein |
| CLIBASIA_00370 |  |  | hypothetical protein |
| CLIBASIA_00465 |  |  | hypothetical protein |
| CLIBASIA_00470 |  |  | hypothetical protein |
| CLIBASIA_00475 |  |  | hypothetical protein |
| CLIBASIA_00480 |  |  | hypothetical protein |
| CLIBASIA_00490 |  |  | hypothetical protein |
| CLIBASIA_00520 |  |  | hypothetical protein |
| CLIBASIA_00525 |  |  | hypothetical protein |
| CLIBASIA_00530 |  |  | hypothetical protein |
| CLIBASIA_00600 |  |  | hypothetical protein |
| CLIBASIA_01000 |  |  | hypothetical protein |
| CLIBASIA_01115 |  |  | hypothetical protein |
| CLIBASIA_01120 |  |  | hypothetical protein |
| CLIBASIA_01195 |  |  | hypothetical protein |
| CLIBASIA_01370 |  |  | hypothetical protein |
| CLIBASIA_01460 |  |  | hypothetical protein |
| CLIBASIA_01660 |  |  | hypothetical protein |
| CLIBASIA_03592 |  |  | hypothetical protein |
| CLIBASIA_03535 |  |  | hypothetical protein |
| CLIBASIA_03362 |  |  | hypothetical protein |
| CLIBASIA_03352 |  |  | hypothetical protein |
| CLIBASIA_03230 |  |  | hypothetical protein |
| CLIBASIA_03090 |  |  | hypothetical protein |
| CLIBASIA_03085 |  |  | hypothetical protein |
| CLIBASIA_02895 |  |  | hypothetical protein |
| CLIBASIA_02890 |  |  | hypothetical protein |
| CLIBASIA_02845 |  |  | hypothetical protein |
| CLIBASIA_02775 |  |  | hypothetical protein |
| CLIBASIA_02770 |  |  | hypothetical protein |
| CLIBASIA_02760 |  |  | hypothetical protein |
| CLIBASIA_02720 |  |  | hypothetical protein |
| CLIBASIA_02715 |  |  | hypothetical protein |
| CLIBASIA_02690 |  |  | hypothetical protein |
| CLIBASIA_02660 |  |  | hypothetical protein |
| CLIBASIA_02655 |  |  | hypothetical protein |
| CLIBASIA_02650 |  |  | hypothetical protein |
| CLIBASIA_02645 |  |  | hypothetical protein |
| CLIBASIA_02640 |  |  | hypothetical protein |
| CLIBASIA_02630 |  |  | hypothetical protein |
| CLIBASIA_02595 |  |  | hypothetical protein |
| CLIBASIA_02590 |  |  | hypothetical protein |
| CLIBASIA_02340 |  |  | hypothetical protein |
| CLIBASIA_02305 |  |  | hypothetical protein |
| CLIBASIA_02275 |  |  | hypothetical protein |
| CLIBASIA_02215 |  |  | hypothetical protein |
| CLIBASIA_02140 |  |  | hypothetical protein |
| CLIBASIA_02025 |  |  | hypothetical protein |
| CLIBASIA_02020 |  |  | DNA-methyltransferase MKpn2kI |
| CLIBASIA_02010 |  |  | hypothetical protein |
| CLIBASIA_02005 |  |  | type II restriction endonuclease |
| CLIBASIA_02000 |  |  | hypothetical protein |
| CLIBASIA_01940 |  |  | hypothetical protein |
| CLIBASIA_03665 |  |  | hypothetical protein |
| CLIBASIA_03670 |  |  | hypothetical protein |
| CLIBASIA_03675 |  |  | hypothetical protein |
| CLIBASIA_03725 |  |  | hypothetical protein |
| CLIBASIA_03730 |  |  | hypothetical protein |
| CLIBASIA_03760 |  |  | hypothetical protein |
| CLIBASIA_03790 |  |  | hypothetical protein |
| CLIBASIA_03875 |  |  | hypothetical protein |
| CLIBASIA_03920 |  |  | hypothetical protein |
| CLIBASIA_03925 |  |  | hypothetical protein |
| CLIBASIA_04025 |  |  | hypothetical protein |
| CLIBASIA_04035 |  |  | hypothetical protein |
| CLIBASIA_04055 |  |  | hypothetical protein |
| CLIBASIA_04250 |  |  | hypothetical protein |
| CLIBASIA_04320 |  |  | hypothetical protein |
| CLIBASIA_04335 |  |  | hypothetical protein |
| CLIBASIA_04405 |  |  | hypothetical protein |
| CLIBASIA_04410 |  |  | hypothetical protein |
| CLIBASIA_04420 |  |  | hypothetical protein |
| CLIBASIA_04430 |  |  | hypothetical protein |
| CLIBASIA_04435 |  |  | hypothetical protein |
| CLIBASIA_04455 |  |  | hypothetical protein |
| CLIBASIA_04475 |  |  | hypothetical protein |
| CLIBASIA_04490 |  |  | hypothetical protein |
| CLIBASIA_04505 |  |  | tRNA (uracil-5-)-methyltransferase |
| CLIBASIA_04510 |  |  | hypothetical protein |
| CLIBASIA_04515 |  |  | hypothetical protein |
| CLIBASIA_04550 |  |  | hypothetical protein |
| CLIBASIA_04560 |  |  | hypothetical protein |
| CLIBASIA_04625 |  |  | hypothetical protein |
| CLIBASIA_04645 |  |  | hypothetical protein |
| CLIBASIA_04650 |  |  | putative Mg2+ chelatase family protein |
| CLIBASIA_04660 |  |  | hypothetical protein |
| CLIBASIA_04690 |  |  | hypothetical protein |
| CLIBASIA_04740 |  |  | hypothetical protein |
| CLIBASIA_04855 |  |  | hypothetical protein |
| CLIBASIA_04860 |  |  | hypothetical protein |
| CLIBASIA_04870 |  |  | hypothetical protein |
| CLIBASIA_04895 |  |  | hypothetical protein |
| CLIBASIA_04915 |  |  | hypothetical protein |
| CLIBASIA_04920 |  |  | hypothetical protein |
| CLIBASIA_04925 |  |  | hypothetical protein |
| CLIBASIA_04930 |  |  | hypothetical protein |
| CLIBASIA_04955 |  |  | hypothetical protein |
| CLIBASIA_04970 |  |  | hypothetical protein |
| CLIBASIA_05105 |  |  | hypothetical protein |
| CLIBASIA_05130 |  |  | hypothetical protein |
| CLIBASIA_05135 |  |  | hypothetical protein |
| CLIBASIA_05145 |  |  | hypothetical protein |
| CLIBASIA_05170 |  |  | hypothetical protein |
| CLIBASIA_05185 |  |  | hypothetical protein |
| CLIBASIA_05210 |  |  | hypothetical protein |
| CLIBASIA_05230 |  |  | hypothetical protein |
| CLIBASIA_05235 |  |  | hypothetical protein |
| CLIBASIA_05240 |  |  | hypothetical protein |
| CLIBASIA_05275 |  |  | hypothetical protein |
| CLIBASIA_05285 |  |  | hypothetical protein |
| CLIBASIA_05305 |  |  | hypothetical protein |
| CLIBASIA_05315 |  |  | hypothetical protein |
| CLIBASIA_05320 |  |  | hypothetical protein |
| CLIBASIA_05330 |  |  | hypothetical protein |
| CLIBASIA_05355 |  |  | hypothetical protein |
| CLIBASIA_05375 |  |  | hypothetical protein |
| CLIBASIA_05435 |  |  | hypothetical protein |
| CLIBASIA_05440 |  |  | hypothetical protein |
| CLIBASIA_05450 |  |  | hypothetical protein |
| CLIBASIA_05455 |  |  | hypothetical protein |
| CLIBASIA_05460 |  |  | hypothetical protein |
| CLIBASIA_05465 |  |  | hypothetical protein |
| CLIBASIA_05570 |  |  | hypothetical protein |
| CLIBASIA_05605 |  |  | hypothetical protein |
| CLIBASIA_05615 |  |  | hypothetical protein |
| CLIBASIA_05650 |  |  | phage associated protein |
| CLIBASIA_05670 |  |  | hypothetical protein |
| CLIBASIA_00445 |  |  | hypothetical protein |
| CLIBASIA_03915 |  |  | hypothetical protein |
| CLIBASIA_04425 |  |  | hypothetical protein |
| CLIBASIA_04885 |  |  | hypothetical protein |
| CLIBASIA_05480 |  |  | hypothetical protein |
| CLIBASIA_04530 |  |  | hypothetical protein |
| CLIBASIA_05140 |  |  | hypothetical protein |
| CLIBASIA_05350 |  |  | hypothetical protein |
| CLIBASIA_02470 | G293_RS03360 |  | hypothetical protein |
| CLIBASIA_04960 | G293_RS00250 |  | hypothetical protein |
| CLIBASIA_04865 | G293_RS05500 |  | hypothetical protein |
| CLIBASIA_04850 | G293_RS00125 |  | hypothetical protein |
| CLIBASIA_05190 | G293_RS05610 |  | hypothetical protein |
| CLIBASIA_01895 |  |  | hypothetical protein |
| CLIBASIA_04315 |  |  | hypothetical protein |
| CLIBASIA_03615 |  |  | hypothetical protein |
| CLIBASIA_04450 |  |  | GCN5-related N-acetyltransferase |
| CLIBASIA_02015 | G293_RS04585 | LAM_RS00815 | type II modification methyltransferase |
| CLIBASIA_03885 |  |  | hypothetical protein |
| CLIBASIA_05310 |  |  | hypothetical protein |
| CLIBASIA_00440 | G293_RS05395 |  | hypothetical protein |
|  | G293_RS04980 |  | hypothetical protein |
|  | G293_RS00060 |  | hypothetical protein |
|  | G293_RS00090 |  | hypothetical protein |
|  | G293_RS00095 |  | hypothetical protein |
|  | G293_RS00100 |  | hypothetical protein |
|  | G293_RS00105 |  | hypothetical protein |
|  | G293_RS00220 |  | hypothetical protein |
|  | G293_RS00235 |  | hypothetical protein |
|  | G293_RS00245 |  | hypothetical protein |
|  | G293_RS00255 |  | hypothetical protein |
|  | G293_RS00285 |  | hypothetical protein |
|  | G293_RS00290 |  | hypothetical protein |
|  | G293_RS00295 |  | hypothetical protein |
|  | G293_RS00370 |  | hypothetical protein |
|  | G293_RS00400 |  | hypothetical protein |
|  | G293_RS00410 |  | hypothetical protein |
|  | G293_RS00445 |  | hypothetical protein |
|  | G293_RS00455 |  | hypothetical protein |
|  | G293_RS00460 |  | hypothetical protein |
|  | G293_RS00480 |  | hypothetical protein |
|  | G293_RS00485 |  | hypothetical protein |
|  | G293_RS00770 |  | hypothetical protein |
|  | G293_RS01065 |  | hypothetical protein |
|  | G293_RS01105 |  | methionyl-tRNA formyltransferase |
|  | G293_RS01185 |  | hypothetical protein |
|  | G293_RS01385 |  | hypothetical protein |
|  | G293_RS01455 |  | hypothetical protein |
|  | G293_RS01580 |  | 3'(2'),5'-bisphosphate nucleotidase CysQ |
|  | G293_RS01655 |  | hypothetical protein |
|  | G293_RS01955 |  | Flp family type IVb pilin |
|  | G293_RS02065 |  | hypothetical protein |
|  | G293_RS02155 |  | hypothetical protein |
|  | G293_RS02165 |  | hypothetical protein |
|  | G293_RS02215 |  | hypothetical protein |
|  | G293_RS02255 |  | hypothetical protein |
|  | G293_RS02280 |  | hypothetical protein |
|  | G293_RS02285 |  | hypothetical protein |
|  | G293_RS02450 |  | hypothetical protein |
|  | G293_RS02845 |  | hypothetical protein |
|  | G293_RS03030 |  | hypothetical protein |
|  | G293_RS03055 |  | hypothetical protein |
|  | G293_RS03120 |  | hypothetical protein |
|  | G293_RS03195 |  | biotin transporter BioY |
|  | G293_RS03200 |  | DUF1284 domain-containing protein |
|  | G293_RS03880 |  | hypothetical protein |
|  | G293_RS03890 |  | hypothetical protein |
|  | G293_RS03895 |  | hypothetical protein |
|  | G293_RS03905 |  | hypothetical protein |
|  | G293_RS03960 |  | hypothetical protein |
|  | G293_RS03985 |  | hypothetical protein |
|  | G293_RS04065 |  | hypothetical protein |
|  | G293_RS04125 |  | hypothetical protein |
|  | G293_RS04195 |  | NADP-dependent malic enzyme |
|  | G293_RS04325 |  | hypothetical protein |
|  | G293_RS04335 |  | hypothetical protein |
|  | G293_RS04340 |  | hypothetical protein |
|  | G293_RS04345 |  | hypothetical protein |
|  | G293_RS04350 |  | hypothetical protein |
|  | G293_RS04430 |  | hypothetical protein |
|  | G293_RS04440 |  | hypothetical protein |
|  | G293_RS04605 |  | hypothetical protein |
|  | G293_RS04975 |  | hypothetical protein |
|  | G293_RS04985 |  | hypothetical protein |
|  | G293_RS04990 |  | hypothetical protein |
|  | G293_RS04995 |  | hypothetical protein |
|  | G293_RS05000 |  | hypothetical protein |
|  | G293_RS05005 |  | hypothetical protein |
|  | G293_RS05010 |  | hypothetical protein |
|  | G293_RS05015 |  | hypothetical protein |
|  | G293_RS05020 |  | hypothetical protein |
|  | G293_RS05025 |  | hypothetical protein |
|  | G293_RS05045 |  | hypothetical protein |
|  | G293_RS05070 |  | hypothetical protein |
|  | G293_RS05285 |  | hypothetical protein |
|  | G293_RS05290 |  | hypothetical protein |
|  | G293_RS05415 |  | hypothetical protein |
|  | G293_RS05440 |  | hypothetical protein |
|  | G293_RS05445 |  | hypothetical protein |
|  | G293_RS05520 |  | DUF1891 domain-containing protein |
|  | G293_RS05555 |  | DUF1153 domain-containing protein |
|  | G293_RS05560 |  | DUF1153 domain-containing protein |
|  | G293_RS05620 |  | UDP-glucose 4-epimerase GalE |
|  | G293_RS05625 |  | UDP-glucose 4-epimerase GalE |
|  | G293_RS05635 |  | phage repressor protein |
|  | G293_RS05650 |  | DUF1153 domain-containing protein |
|  |  | LAM_RS00810 | hypothetical protein |
|  |  | LAM_RS00985 | DNA-3-methyladenine glycosylase I |
|  |  | LAM_RS01525 | hypothetical protein |
|  |  | LAM_RS01820 | hypothetical protein |
|  |  | LAM_RS01830 | hypothetical protein |
|  |  | LAM_RS02050 | hypothetical protein |
|  |  | LAM_RS02405 | hypothetical protein |
|  |  | LAM_RS05145 | lipid A ABC exporter family |
|  |  | LAM_RS02720 | hypothetical protein |
|  |  | LAM_RS02885 | hypothetical protein |
|  |  | LAM_RS02940 | hypothetical protein |
|  |  | LAM_RS02945 | hypothetical protein |
|  |  | LAM_RS05160 | IS630 family transposase |
|  |  | LAM_RS03200 | hypothetical protein |
|  |  | LAM_RS03230 | hypothetical protein |
|  |  | LAM_RS03240 | hypothetical protein |
|  |  | LAM_RS03250 | hypothetical protein |
|  |  | LAM_RS03265 | hypothetical protein |
|  |  | LAM_RS03270 | hypothetical protein |
|  |  | LAM_RS03320 | hypothetical protein |
|  |  | LAM_RS03350 | hypothetical protein |
|  |  | LAM_RS03355 | hypothetical protein |
|  |  | LAM_RS03435 | hypothetical protein |
|  |  | LAM_RS03450 | hypothetical protein |
|  |  | LAM_RS03680 | hypothetical protein |
|  |  | LAM_RS03685 | hypothetical protein |
|  |  | LAM_RS03695 | hypothetical protein |
|  |  | LAM_RS04045 | hypothetical protein |
|  |  | LAM_RS04095 | hypothetical protein |
|  |  | LAM_RS04145 | hypothetical protein |
|  |  | LAM_RS04675 | hypothetical protein |
|  |  | LAM_RS04720 | hypothetical protein |
|  |  | LAM_RS04725 | hypothetical protein |
|  |  | LAM_RS04730 | hypothetical protein |
|  |  | LAM_RS04820 | hypothetical protein |
|  |  | LAM_RS05115 | hypothetical protein |
|  |  | LAM_RS01825 | hypothetical protein |
|  |  | LAM_RS03235 | hypothetical protein |
|  |  | LAM_RS03215 | hypothetical protein |
|  |  | LAM_RS01835 | hypothetical protein |
|  |  | LAM_RS03600 | hypothetical protein |
|  |  | LAM_RS04440 | hypothetical protein |
