# Supplementary material for: Genome‐wide analyses of Liberibacter species provides insights into evolution, phylogenetic relationships, and virulence factors
Source: Mol Plant Pathol. 2020 Feb 28;21(5):716–31. doi: 10.1111/mpp.12925 (PMC7170780; doi:10.1111/mpp.12925)
Supplement: Supplementary file 14 [file MPP-21-716-s014.docx]

Table S7. Genes present Liberibacter crescens (LC_BT1), *Candidatus* Liberibacter solanacearum (Lso_ZC1), and *Ca.* Liberibacter europaeus (Leu_ASNZ1), but absent in Huanglongbing (HLB) associated Liberibacter isolates.

| **Lc_BT1** | **Lso_ZC1** | **Leu_ASNZ1** |
| --- | --- | --- |
| B488_RS00070 | CMC_RS02790 | C4617_00645 |
| B488_RS00075 | CMC_RS02920 | C4617_01170 |
| B488_RS00085 | CMC_RS04235 | C4617_01175 |
| B488_RS00100 | CMC_RS04955 | C4617_01185 |
| B488_RS06755 | CMC_RS05515 | C4617_01190 |
| B488_RS00105 | CMC_RS05895 | C4617_01205 |
| B488_RS00130 | CMC_RS00050 | C4617_01320 |
| B488_RS00135 | CMC_RS00070 | C4617_02120 |
| B488_RS00165 | CMC_RS00145 | C4617_02260 |
| B488_RS00215 | CMC_RS01855 | C4617_02285 |
| B488_RS00270 | CMC_RS01910 | C4617_02610 |
| B488_RS00310 | CMC_RS02020 | C4617_02705 |
| B488_RS00315 | CMC_RS02050 | C4617_02790 |
| B488_RS06760 | CMC_RS05930 | C4617_02860 |
| B488_RS00365 | CMC_RS02710 | C4617_02885 |
| B488_RS00370 | CMC_RS02720 | C4617_02890 |
| B488_RS00415 | CMC_RS02785 | C4617_02960 |
| B488_RS00420 | CMC_RS03015 | C4617_03045 |
| B488_RS00430 | CMC_RS03020 | C4617_03170 |
| B488_RS00460 | CMC_RS05805 | C4617_03195 |
| B488_RS00500 | CMC_RS03030 | C4617_03200 |
| B488_RS00505 | CMC_RS03290 | C4617_03240 |
| B488_RS00510 | CMC_RS03310 | C4617_03270 |
| B488_RS06965 | CMC_RS03375 | C4617_03395 |
| B488_RS00575 | CMC_RS03405 | C4617_03410 |
| B488_RS00595 | CMC_RS03425 | C4617_03575 |
| B488_RS00605 | CMC_RS03440 | C4617_03585 |
| B488_RS00615 | CMC_RS03490 | C4617_03610 |
| B488_RS00680 | CMC_RS03495 | C4617_03615 |
| B488_RS00700 | CMC_RS03500 | C4617_03865 |
| B488_RS00720 | CMC_RS03855 | C4617_03895 |
| B488_RS00725 | CMC_RS03875 | C4617_03910 |
| B488_RS00730 | CMC_RS03880 | C4617_03995 |
| B488_RS00735 | CMC_RS05975 | C4617_04000 |
| B488_RS00760 | CMC_RS05980 | C4617_04135 |
| B488_RS00775 | CMC_RS04265 | C4617_04140 |
| B488_RS00815 | CMC_RS04360 | C4617_04145 |
| B488_RS00840 | CMC_RS06000 | C4617_04185 |
| B488_RS00940 | CMC_RS04980 | C4617_04275 |
| B488_RS00945 | CMC_RS04985 | C4617_04300 |
| B488_RS00950 | CMC_RS05385 | C4617_04385 |
| B488_RS00955 | CMC_RS06015 | C4617_04390 |
| B488_RS00960 | CMC_RS06020 | C4617_04430 |
| B488_RS00965 | CMC_RS05520 | C4617_04435 |
| B488_RS00970 | CMC_RS05620 | C4617_04445 |
| B488_RS06975 | CMC_RS00870 | C4617_04455 |
| B488_RS01090 | CMC_RS00885 | C4617_04460 |
| B488_RS01095 | CMC_RS00945 | C4617_04495 |
| B488_RS01130 | CMC_RS00965 | C4617_04500 |
| B488_RS01140 | CMC_RS00970 | C4617_04510 |
| B488_RS01155 | CMC_RS00990 | C4617_04520 |
| B488_RS01230 | CMC_RS05785 | C4617_04525 |
| B488_RS01240 | CMC_RS01005 | C4617_04585 |
| B488_RS01250 | CMC_RS01010 | C4617_04660 |
| B488_RS01390 | CMC_RS03350 | C4617_04665 |
| B488_RS01455 | CMC_RS03355 | C4617_04680 |
| B488_RS01460 | CMC_RS03365 | C4617_04730 |
| B488_RS01480 | CMC_RS05510 | C4617_04740 |
| B488_RS01485 | CMC_RS05550 | C4617_04780 |
| B488_RS01510 | CMC_RS05565 | C4617_04785 |
| B488_RS01535 | CMC_RS05645 | C4617_04790 |
| B488_RS01545 | CMC_RS05660 | C4617_04800 |
| B488_RS01590 | CMC_RS05665 | C4617_04805 |
| B488_RS01635 | CMC_RS05685 | C4617_04830 |
| B488_RS01645 | CMC_RS05860 | C4617_04915 |
| B488_RS01670 | CMC_RS05700 | C4617_05040 |
| B488_RS01675 | CMC_RS05705 | C4617_05045 |
| B488_RS01690 | CMC_RS03385 | C4617_05080 |
| B488_RS01740 | CMC_RS03390 | C4617_05245 |
| B488_RS01780 | CMC_RS03400 | C4617_05310 |
| B488_RS01785 | CMC_RS03360 | C4617_05350 |
| B488_RS01825 | CMC_RS00985 | C4617_05355 |
| B488_RS01875 | CMC_RS00975 | C4617_05360 |
| B488_RS01900 | CMC_RS03395 | C4617_05445 |
| B488_RS01905 | CMC_RS05680 | C4617_05450 |
| B488_RS01945 | CMC_RS05670 | C4617_05490 |
| B488_RS01950 | CMC_RS03420 | C4617_05500 |
| B488_RS01955 | CMC_RS00035 | C4617_05525 |
| B488_RS01960 | CMC_RS02795 | C4617_05555 |
| B488_RS02145 | CMC_RS04460 | C4617_05760 |
| B488_RS02170 | CMC_RS04950 | C4617_05765 |
| B488_RS02175 | CMC_RS03175 | C4617_05770 |
| B488_RS02220 | CMC_RS03180 | C4617_05825 |
| B488_RS02260 | CMC_RS03915 | C4617_05830 |
| B488_RS02275 | CMC_RS05810 | C4617_05840 |
| B488_RS02280 | CMC_RS05945 | C4617_00035 |
| B488_RS02290 |  | C4617_03280 |
| B488_RS02295 |  | C4617_03370 |
| B488_RS02300 |  | C4617_03680 |
| B488_RS02305 |  | C4617_03915 |
| B488_RS02320 |  | C4617_04450 |
| B488_RS02335 |  | C4617_04505 |
| B488_RS02340 |  | C4617_04750 |
| B488_RS02350 |  | C4617_05540 |
| B488_RS02355 |  | C4617_05050 |
| B488_RS02360 |  | C4617_04255 |
| B488_RS02365 |  | C4617_05240 |
| B488_RS02370 |  | C4617_05505 |
| B488_RS02385 |  | C4617_04770 |
| B488_RS02390 |  | C4617_05560 |
| B488_RS02395 |  | C4617_03300 |
| B488_RS02400 |  | C4617_03400 |
| B488_RS02415 |  | C4617_03620 |
| B488_RS02420 |  | C4617_04125 |
| B488_RS02430 |  | C4617_04530 |
| B488_RS02445 |  | C4617_04795 |
| B488_RS02450 |  | C4617_04835 |
| B488_RS02460 |  | C4617_05470 |
| B488_RS02465 |  | C4617_05325 |
| B488_RS02470 |  | C4617_04310 |
| B488_RS02475 |  | C4617_05330 |
| B488_RS02495 |  | C4617_04685 |
| B488_RS02500 |  | C4617_05075 |
| B488_RS02510 |  | C4617_02785 |
| B488_RS02520 |  | C4617_03405 |
| B488_RS02575 |  | C4617_05465 |
| B488_RS02605 |  | C4617_02710 |
| B488_RS02615 |  | C4617_02880 |
| B488_RS02650 |  | C4617_04400 |
| B488_RS02655 |  | C4617_05340 |
| B488_RS02730 |  | C4617_05610 |
| B488_RS02770 |  | C4617_03570 |
| B488_RS02790 |  |  |
| B488_RS02805 |  |  |
| B488_RS02815 |  |  |
| B488_RS02820 |  |  |
| B488_RS02855 |  |  |
| B488_RS02870 |  |  |
| B488_RS02875 |  |  |
| B488_RS02880 |  |  |
| B488_RS02885 |  |  |
| B488_RS02960 |  |  |
| B488_RS02965 |  |  |
| B488_RS03045 |  |  |
| B488_RS03065 |  |  |
| B488_RS03135 |  |  |
| B488_RS03140 |  |  |
| B488_RS03175 |  |  |
| B488_RS03180 |  |  |
| B488_RS03185 |  |  |
| B488_RS03190 |  |  |
| B488_RS03215 |  |  |
| B488_RS06845 |  |  |
| B488_RS03240 |  |  |
| B488_RS03245 |  |  |
| B488_RS03250 |  |  |
| B488_RS03255 |  |  |
| B488_RS03270 |  |  |
| B488_RS03275 |  |  |
| B488_RS03280 |  |  |
| B488_RS07015 |  |  |
| B488_RS03290 |  |  |
| B488_RS03310 |  |  |
| B488_RS03350 |  |  |
| B488_RS03405 |  |  |
| B488_RS03410 |  |  |
| B488_RS03420 |  |  |
| B488_RS03510 |  |  |
| B488_RS03515 |  |  |
| B488_RS07025 |  |  |
| B488_RS03525 |  |  |
| B488_RS03530 |  |  |
| B488_RS03555 |  |  |
| B488_RS03565 |  |  |
| B488_RS06860 |  |  |
| B488_RS03670 |  |  |
| B488_RS03685 |  |  |
| B488_RS03690 |  |  |
| B488_RS03695 |  |  |
| B488_RS03705 |  |  |
| B488_RS03760 |  |  |
| B488_RS03780 |  |  |
| B488_RS03785 |  |  |
| B488_RS03845 |  |  |
| B488_RS03855 |  |  |
| B488_RS07030 |  |  |
| B488_RS03880 |  |  |
| B488_RS03885 |  |  |
| B488_RS06880 |  |  |
| B488_RS03920 |  |  |
| B488_RS03955 |  |  |
| B488_RS03980 |  |  |
| B488_RS03985 |  |  |
| B488_RS03990 |  |  |
| B488_RS03995 |  |  |
| B488_RS04000 |  |  |
| B488_RS04005 |  |  |
| B488_RS04015 |  |  |
| B488_RS04080 |  |  |
| B488_RS04115 |  |  |
| B488_RS04125 |  |  |
| B488_RS04150 |  |  |
| B488_RS04280 |  |  |
| B488_RS04300 |  |  |
| B488_RS04305 |  |  |
| B488_RS04310 |  |  |
| B488_RS04330 |  |  |
| B488_RS04335 |  |  |
| B488_RS04370 |  |  |
| B488_RS04405 |  |  |
| B488_RS04410 |  |  |
| B488_RS04415 |  |  |
| B488_RS04465 |  |  |
| B488_RS04545 |  |  |
| B488_RS04600 |  |  |
| B488_RS04655 |  |  |
| B488_RS04675 |  |  |
| B488_RS04690 |  |  |
| B488_RS04695 |  |  |
| B488_RS04700 |  |  |
| B488_RS04780 |  |  |
| B488_RS04800 |  |  |
| B488_RS04805 |  |  |
| B488_RS04810 |  |  |
| B488_RS04860 |  |  |
| B488_RS06915 |  |  |
| B488_RS04885 |  |  |
| B488_RS04940 |  |  |
| B488_RS04970 |  |  |
| B488_RS04995 |  |  |
| B488_RS05025 |  |  |
| B488_RS05035 |  |  |
| B488_RS05045 |  |  |
| B488_RS05055 |  |  |
| B488_RS05060 |  |  |
| B488_RS05080 |  |  |
| B488_RS05105 |  |  |
| B488_RS05110 |  |  |
| B488_RS05150 |  |  |
| B488_RS05170 |  |  |
| B488_RS06920 |  |  |
| B488_RS06925 |  |  |
| B488_RS05215 |  |  |
| B488_RS05310 |  |  |
| B488_RS05385 |  |  |
| B488_RS05390 |  |  |
| B488_RS06940 |  |  |
| B488_RS05400 |  |  |
| B488_RS05410 |  |  |
| B488_RS05450 |  |  |
| B488_RS05465 |  |  |
| B488_RS05470 |  |  |
| B488_RS05490 |  |  |
| B488_RS05510 |  |  |
| B488_RS05535 |  |  |
| B488_RS05545 |  |  |
| B488_RS05555 |  |  |
| B488_RS05565 |  |  |
| B488_RS05650 |  |  |
| B488_RS05655 |  |  |
| B488_RS05660 |  |  |
| B488_RS05665 |  |  |
| B488_RS05680 |  |  |
| B488_RS05750 |  |  |
| B488_RS06950 |  |  |
| B488_RS05755 |  |  |
| B488_RS05770 |  |  |
| B488_RS05820 |  |  |
| B488_RS05835 |  |  |
| B488_RS05890 |  |  |
| B488_RS05900 |  |  |
| B488_RS05910 |  |  |
| B488_RS05940 |  |  |
| B488_RS05945 |  |  |
| B488_RS05965 |  |  |
| B488_RS05975 |  |  |
| B488_RS05980 |  |  |
| B488_RS05990 |  |  |
| B488_RS05995 |  |  |
| B488_RS06015 |  |  |
| B488_RS06030 |  |  |
| B488_RS06035 |  |  |
| B488_RS06040 |  |  |
| B488_RS06060 |  |  |
| B488_RS06070 |  |  |
| B488_RS06085 |  |  |
| B488_RS06100 |  |  |
| B488_RS06105 |  |  |
| B488_RS06110 |  |  |
| B488_RS06120 |  |  |
| B488_RS06205 |  |  |
| B488_RS06210 |  |  |
| B488_RS06220 |  |  |
| B488_RS06225 |  |  |
| B488_RS06270 |  |  |
| B488_RS06275 |  |  |
| B488_RS06280 |  |  |
| B488_RS06320 |  |  |
| B488_RS06360 |  |  |
| B488_RS06365 |  |  |
| B488_RS06375 |  |  |
| B488_RS06380 |  |  |
| B488_RS06405 |  |  |
| B488_RS06470 |  |  |
| B488_RS06490 |  |  |
| B488_RS06500 |  |  |
| B488_RS06530 |  |  |
| B488_RS07060 |  |  |
| B488_RS06535 |  |  |
| B488_RS06540 |  |  |
| B488_RS06545 |  |  |
| B488_RS06550 |  |  |
| B488_RS06600 |  |  |
| B488_RS06605 |  |  |
| B488_RS06620 |  |  |
| B488_RS06640 |  |  |
| B488_RS06645 |  |  |
| B488_RS06650 |  |  |
| B488_RS06655 |  |  |
| B488_RS06695 |  |  |
| B488_RS06725 |  |  |
| B488_RS00320 |  |  |
| B488_RS00480 |  |  |
| B488_RS00670 |  |  |
| B488_RS06980 |  |  |
| B488_RS04345 |  |  |
| B488_RS04340 |  |  |
| B488_RS06840 |  |  |
| B488_RS03070 |  |  |
| B488_RS06890 |  |  |
| B488_RS03055 |  |  |
| B488_RS04265 |  |  |
| B488_RS06825 |  |  |
| B488_RS03160 |  |  |
| B488_RS06515 |  |  |
| B488_RS05970 |  |  |
| B488_RS06615 |  |  |
| B488_RS00470 |  |  |
| B488_RS00660 |  |  |
| B488_RS06370 |  |  |
| B488_RS02270 |  |  |
| B488_RS02285 |  |  |
| B488_RS06800 |  |  |
| B488_RS02380 |  |  |
| B488_RS02425 |  |  |
| B488_RS02435 |  |  |
| B488_RS02525 |  |  |
| B488_RS02700 |  |  |
| B488_RS03155 |  |  |
| B488_RS04785 |  |  |
| B488_RS04965 |  |  |
| B488_RS06610 |  |  |
| B488_RS00665 |  |  |
| B488_RS02375 |  |  |
| B488_RS06810 |  |  |
| B488_RS02740 |  |  |
| B488_RS03035 |  |  |
| B488_RS00675 |  |  |
| B488_RS04285 |  |  |
| B488_RS06815 |  |  |
| B488_RS06520 |  |  |
| B488_RS04315 |  |  |
| B488_RS05475 |  |  |
| B488_RS06895 |  |  |
| B488_RS06820 |  |  |
| B488_RS06525 |  |  |
| B488_RS07005 |  |  |
| B488_RS02325 |  |  |
| B488_RS02330 |  |  |
| B488_RS02405 |  |  |
| B488_RS03030 |  |  |
| B488_RS00080 |  |  |
| B488_RS03050 |  |  |
| B488_RS05600 |  |  |
| B488_RS00385 |  |  |
| B488_RS03850 |  |  |
| B488_RS06795 |  |  |
| B488_RS03930 |  |  |
| B488_RS04325 |  |  |
| B488_RS06830 |  |  |
| B488_RS06095 |  |  |
| B488_RS06790 |  |  |
| B488_RS06835 |  |  |
| B488_RS01620 |  |  |
| B488_RS06635 |  |  |
| B488_RS06805 |  |  |
| B488_RS06900 |  |  |
| B488_RS02635 |  |  |
| B488_RS02680 |  |  |
| B488_RS06985 |  |  |
| B488_RS06990 |  |  |
| B488_RS06995 |  |  |
| B488_RS07000 |  |  |
| B488_RS04400 |  |  |
| B488_RS01625 |  |  |
